# Eco-evolutionary dynamics lead to functionally robust and redundant communities

**DOI:** 10.1371/journal.pcbi.1014437

**Published:** 2026-07-21

**Authors:** Lorenzo Fant, Iuri Macocco, Jacopo Grilli

**Affiliations:** 1 Dynamics of Ecosystems and Computational Oceanography (ECHO), National Institute of Oceanography and Applied Geophysics - OGS, Trieste, Italy; 2 National Biodiversity Future Centre - NBFC, Università di Palermo, Palermo, Italy; 3 International School for Advanced Studies - SISSA, Trieste, Italy; 4 Department of Translation and Language Sciences, Universitat Pompeu Fabra, Barcelona, Spain; 5 Quantitative Life Sciences, The Abdus Salam International Centre for Theoretical Physics, Trieste, Italy; National Taiwan University, TAIWAN

## Abstract

Microbial communities are taxonomically diverse and variable: species presence and abundances widely fluctuate over time, space, and even across biological replicates under controlled experimental conditions. However, environmental conditions exert strong selection on the traits of community members and their functions. Similar environmental conditions are expected to produce functionally similar communities. This environmental selection, combined with taxonomic variability, leads to the influential concept of functional redundancy — the idea that many species can perform the same function, allowing communities with different species compositions to maintain identical functional profiles. Despite the centrality of functional redundancy in microbial ecology, we lack a theoretical understanding of its origin. Here we study the eco-evolutionary dynamics of communities interacting through competition and cross-feeding. We show that eco-evolutionary trajectories rapidly converge to a “functional attractor” — a functional composition uniquely determined by environmental conditions. Taxonomic composition follows non-reproducible dynamics while being constrained by the conservation of functional composition. Our framework provides a theoretical foundation for understanding functional robustness and redundancy in microbial communities.

## Author summary

Microbial communities harbor thousands of species, and their taxonomic composition varies greatly across samples and over time. Yet, a growing body of evidence suggests that the collective functions performed by these communities are remarkably stable. This phenomenon, known as functional redundancy, implies that different species can substitute for one another without changing what the community does. Despite its importance, we lack a theoretical explanation for how functional redundancy arises. Here, we develop a mathematical model that couples ecological dynamics (competition for resources, cross-feeding of metabolic by-products) with evolutionary processes (mutations in metabolic capabilities and fitness). We show that, under a metabolic trade-off where consuming more resources incurs a higher cost, communities rapidly converge to a stable “functional attractor” determined solely by resource availability. While the identity and abundance of individual species continue to change, the overall functional profile remains fixed. Our results provide a mechanistic explanation for the widely observed pattern of functional redundancy and identify the conditions under which it holds or breaks down.

## Introduction

One of the most fascinating aspects of microbial communities is their taxonomic diversity and variability. Thousands of species populate a gram of soil, and another gram collected just one meter away would have a different species composition [1]. This observation spans virtually all environments: from glaciers to oceans, from grasslands to guts.

This variability observed in natural communities is quantitatively similar across environments [2] and shows similar characteristics when measured between communities and over time [3]. The magnitude of this variability persists across a wide range of taxonomic resolutions [4,5] and in microcosm experiments under controlled conditions [6–8].

Although taxonomic composition is consistently variable, microbial communities are often clearly organized in terms of trophic levels, functional composition, or traits [9]. These functional patterns can even become visible to the naked eye in the presence of gradients, such as oxygen and sulfur gradients in Winogradsky columns or temperature gradients in hot springs. A trait-based description [9] is therefore more natural in these contexts, though it requires assuming a priori which traits the environment selects for reproducibility.

The coexistence of taxonomic variability and replicable functional organization led to the influential concept of functional redundancy [10–12]. This concept posits that since many species can perform the same functions, multiple species combinations can correspond to the same functional profile. Thus, taxonomic variability would mask the underlying functional robustness.

The evidence for functional redundancy and robustness is scattered, indirect, and mostly qualitative. Comparisons between taxonomic abundances and gene family abundances from metagenomic data (used as proxies of functional composition) show different levels of variation [11,12], with gene families being more stable and taxa widely fluctuating. In experimental communities, abundance at coarse taxonomic levels (used as a proxy for similar functional profiles) is much less variable than at fine taxonomic levels [6]. However, these comparisons are mostly qualitative and it is debated whether the results reflect robust biological processes or are statistically null [13].

Consumer-resource models are the main modeling framework for microbial communities. Their origin goes back to the classic work of MacArthur and Levins [14], which has been extensively studied and discussed in the following decades [15,16], mostly to describe the coexistence of a handful of species. These models have been further extended to consider facilitation through cross-feeding [6,17,18], where species change resource availability not only by consumption, but also because they release into the environment the waste products of their metabolism. These models qualitatively describe experimental results [6,19] and have the flexibility to reproduce patterns observed in empirical microbial communities [20].

Once the parameters of the model — such as consumers’ resource preferences and resources supply rates — are set and an initial pool of species is chosen, populations converge, over large enough times, to an equilibrium point. Under some mild conditions, identified over decades of theoretical work [21,22], consumer-resource models are characterized by a globally stable equilibrium: the steady state is independent of the initial population abundances and resource concentrations. The competitive exclusion principle — one of the most fundamental results of theoretical ecology — limits the number of species that can coexist in a stable equilibrium: diversity cannot exceed the number of resources. While this bound is hard, it is often not realized, as only fewer species can coexist [23,24].

The number and identity of the species coexisting at equilibrium are in fact determined not only by the ecological dynamics, but also by the initial pool of species. This initial pool of species is often interpreted as the metacommunity diversity: the ecological dynamics unfold in a local community which is coupled to the metacommunity by rare migrations. Most of the recent progress in understanding the assembly of large ecological communities has been driven by the assumption of random species pools [24–26]. This choice assumes that the parameters characterizing species’ physiological and ecological traits are independently drawn from some distribution. This assumption implicitly underlies a separation of spatial and temporal scales: the ecological dynamics determining the community composition in the local community occur independently of the evolutionary processes determining the pool of diversity of the metacommunity.

Instead of assuming a fixed species pool, one can allow individual traits to evolve dynamically, including the ones specifying their interactions with other individuals and the environment. Classic work in adaptive dynamics [27] has shown how, starting from a clonal population, diversification can evolve under general conditions of frequency-dependent selection. Several works have then studied eco-evolutionary dynamics of interacting populations [28,29], by allowing individuals’ traits to be subject to mutations and to be inherited by the following generation. “Intrinsic” fitness (how fast populations grow in an optimal environment) and niche differences (how the growth of different populations is coupled) both influence community evolution, and it is their interplay that determines the observed diversity of an evolved community [30].

A key difficulty in interpreting the outcomes of eco-evolutionary dynamics is the fact that there are no natural degrees of freedom to characterize the evolution of the community. The identity of populations, and not only their abundance, is under constant change. Here we show that the functional composition emerges as the natural variable that characterizes the composition of the community. In the setting of consumer-resource models, we identify the “function” as the individual ability to consume a resource and the “functional profile” as the fraction of individuals able to consume a given resource. This definition is natural in the context of community, functional, and trait-based ecology, and also provides a natural connection with the observations stemming from metagenomic studies, as the ability to grow on a given resource can be associated with the presence or absence of a given gene.

It is important to notice that this interpretation of “function” is not the only possible or interesting one. In particular, “function” is often used to refer to the services that microbial communities perform for their host or the environment. These functions cannot necessarily be reduced to a property of an individual cell, but they are rather emergent features of a community (e.g., the gut health of a host).

We consider the broad framework of consumer-resource-crossfeeding models under an explicit eco-evolutionary dynamics, where strains differ in their resource preferences and their intrinsic fitness. Higher resource intakes are balanced by lower efficiency (or equivalently, higher mortality) implemented by a metabolic trade-off [31–33]. While previous studies have characterized the ecological equilibria of consumer-resource models with fixed species pools [31,32], our work introduces explicit evolutionary dynamics — mutations and horizontal gene transfer — and demonstrates that the eco-evolutionary trajectories converge to the same functional attractor predicted by the infinite-pool limit. We analytically predict the stationary functional composition — here defined as the fraction of individuals able to grow on a given resource — and validate these predictions against the full eco-evolutionary simulations, including in the presence of cross-feeding. Interestingly, we show that, once the functional attractor is reached, the strain dynamics are then dominated by fitness differences, implying that functional composition is robust (independent of small fitness differences) and redundant (achieved under multiple strain compositions). This two-phase dynamics — fast functional convergence followed by slow fitness-driven taxonomic turnover — provides a mechanistic explanation for functional redundancy in microbial communities.

## Results

### Consumer-resource-crossfeeding model with metabolic tradeoff

**Fig 1:**
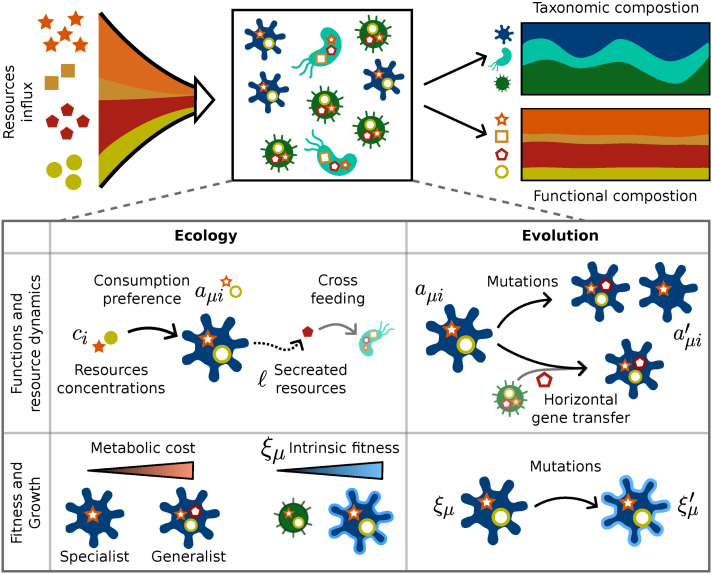
Eco-evolutionary dynamics in consumer-resource communities with cross-feeding and metabolic trade-offs. The model integrates ecological competition for resources with evolutionary processes to explain functional redundancy in microbial communities. Community composition (top): While taxonomic composition exhibits high variability and turnover over time, functional composition $F_{i}$ (fraction of individuals consuming each resource) converges to a stable, predictable attractor independent of strain identity. This can be interpreted as functional redundancy emerging from the interplay between ecological selection pressures and evolutionary innovation, allowing multiple taxonomic configurations to achieve identical functional profiles. Ecology (bottom left): Strains with consumption preferences $a_{\mu i}$ compete for resources with concentrations $c_{i}$ in an environment with cross-feeding, where fraction ℓ of consumed resources is converted and released as different metabolites. A metabolic trade-off penalizes generalists (consuming many resources) with reduced growth efficiency compared to specialists, while strains also differ in intrinsic fitness $\xi_{\mu }$ independent of their resource consumption abilities. Evolution (bottom right): Two types of mutations alter consumption preferences: spontaneous mutations that randomly change resource utilization capabilities, and horizontal gene transfer that allows acquisition of consumption traits proportional to their frequency $F_{i}$ in the community. Intrinsic fitness values $\xi_{\mu }$ also mutate independently.

Our ecological framework builds on the standard consumer-resource-crossfeeding model [17] that describes how microbial populations interact with their environment through resource consumption and metabolic exchange (see Fig 1). The per-capita growth rate of a strain μ is given by:


$$\begin{array}{c} g_{\mu }(\underset{―}{c})=\eta_{\mu }\left((1-\ell )\sum_{i=1}^{R}a_{\mu i}r_{i}(c_{i})-\frac{1}{\tau }H\left(\chi \sum_{i}a_{\mu i}\right)\left(1-\xi_{\mu }\right)\right) \end{array}$$ (1)

This equation captures several key biological assumptions. Individuals are characterized by their resource preferences $a_{\mu i}$, which determine how efficiently they can consume each resource: $a_{\mu i}=0$ means no consumption of resource *i*, while $a_{\mu i}=1$ represents maximum consumption efficiency. The functional form $r_{i}(c_{i})$ encodes the dependency of the growth rate on the concentration $c_{i}$ of resource *i* (also known as functional response). We consider both linear and saturating (Monod-like) functional dependencies (see Materials and methods).

A key assumption in our approach is the metabolic trade-off implemented through the term $H\left(\chi \sum_{i}a_{\mu i}\right)$, which is a monotonically increasing function of the sum of consumption rates $\sum_{i}a_{\mu i}$. This captures the cost of consuming more resources. The form of the trade-off generalizes the case considered in [31,32], which assumes a constant total energy budget devoted to metabolism (*H*(*z*)=*z*).

While in the Materials and Methods we show that our results hold for linear, sub-linear, and super-linear tradeoffs, in the main text we explicitly use the linear case *H*(*z*)=1 + *z*. When χ=0, there is no trade-off and generalists face no disadvantage. As χ increases, the cost of metabolic versatility becomes more severe. The fixed energy budget scenario [31,32] (*H*(*z*)=*z*) is recovered in the limit of large values of χ.

Resource dynamics are modeled explicitly, incorporating both consumption and cross-feeding. Resources are externally supplied at constant rates $h_{i}$ and consumed by populations proportionally to their preferences. A fraction (1−ℓ) of consumed resources is used for growth, while the remaining fraction ℓ is converted into different metabolites and released back into the environment. This cross-feeding process, described by a transformation matrix $D_{ij}$, allows populations to indirectly benefit from resources they cannot directly consume [17]. Specifically, the concentration $c_{i}$ of resource *i* evolves according to


$$\begin{array}{c} \frac{dc_{i}}{dt}=h_{i}-\sum_{\sigma }n_{\sigma }a_{\sigma i}r_{i}(c_{i})+\sum_{j}D_{ij}\ell \sum_{\sigma }n_{\sigma }a_{\sigma j}r_{j}(c_{j})\;, \end{array}$$ (2)

where the first term represents the external supply of resources, the second term represents consumption, and the third captures cross-feeding (see Materials and methods for full details).

The parameter $\xi_{\mu }$ represents intrinsic fitness differences. These are physiological variations that modulate the maintenance cost of an individual, independent of its resource utilization pattern. In the per-capita growth rate (Eq. 1), $\xi_{\mu }$ enters as a multiplicative factor $\left(1-\xi_{\mu }\right)$ on the death rate. This is mathematically equivalent to a fitness effect on the net growth rate (see Materials and methods). Such differences, which we refer to as intrinsic fitness, determine which population survives when two populations with identical resource preferences are competing. We use the term “strain” to identify groups of individuals sharing both identical resource preferences and intrinsic fitness values, while “ecotype” refers to strains with the same resource preferences but potentially different intrinsic fitness.

### Evolution of resource preferences and intrinsic fitness

The evolutionary component of our model allows both resource preferences and intrinsic fitness to change through mutation. This dual evolution captures two distinct types of genetic changes observed in microbial populations: those affecting ecologically relevant phenotypes (such as losing the ability to transport a specific sugar) and those affecting general cellular processes (such as changes in ribosome efficiency) [30].

Mutations affecting resource preferences follow biologically motivated rules. The rate at which a strain loses the ability to consume a resource is constant, reflecting the general tendency for unused metabolic capabilities to be lost. Conversely, the rate of gaining new metabolic capabilities depends on two mechanisms: horizontal gene transfer, where the acquisition rate is proportional to how common that capability is in the population, and de novo mutations, which occur at a constant background rate. We consider different implementations of the mutational steps (e.g., including different scenarios for the relative rate of horizontal gene transfer, see Materials and methods) which, however, do not affect the results that are presented in the following.

Intrinsic fitness mutations are modeled as small random changes. We consider two radically different implementations of fitness evolution. As a first scenario, We consider the new value of the intrinsic fitness is drawn at random from a fixed distribution of width ϵ, independently of the parent’s fitness value. To ensure that the intrinsic fitness does not evolve directionally we extract a random value using the phenotype as a seed of the generator. This guarantees that the intrinsic fitness does not evolve towards the tails of the distribution by multiple extractions of the same species. The parameter ϵ controls the magnitude of fitness differences between strains, allowing us to explore scenarios ranging from nearly neutral evolution (small ϵ) to strongly selective regimes (large ϵ). We focus on the case of small fitness differences and extensively explore the effect of increasing ϵ values (see Materials and methods). This scenario is consistent with the biological interpretation that fitness differences arise from context-dependent factors (e.g., phage susceptibility, temperature adaptation) rather than from progressively optimizable traits [30]. This latter case corresponds to the second scenario, where we allowed $\xi_{\mu }$ to evolve as in a a staircase model, which mutations leading to larger fitness values without bound (see Materials and methods). Fig A in S1 Appendix shows that the second scenario leads to the same results.

The eco-evolutionary dynamics unfold as a sequence of ecological equilibration followed by successful invasions. When a new mutant appears, it faces demographic stochasticity during its initial growth phase. We account for this by calculating survival probabilities based on the mutant’s growth rate in the current environment (see Materials and methods). Only mutants that survive this bottleneck can potentially invade and alter the community composition.

This framework allows us to study how functional composition emerges and stabilizes even as the taxonomic composition of communities continues to evolve. The interplay between ecological selection (favoring efficient resource utilization and higher intrinsic fitness) and evolutionary constraints (the metabolic trade-off) drives the eco-evolutionary dynamics.

### Eco-evolutionary dynamics produce stable functional attractors despite strain-level variability

**Fig 2:**
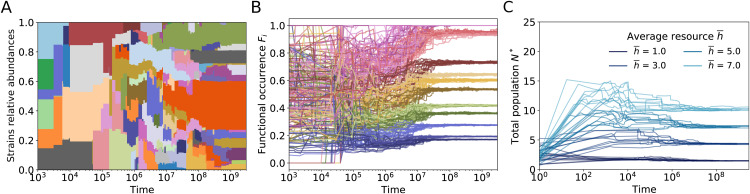
Stability of functional occurrences $F_{i}$ for communities evolving under a consumer-resource model. The system is initialized with a small number of initial random strains, chosen so that each gene is present at least once. The system evolves in a chemostat with fixed resources input. When equilibrium is reached, one mutant is added to the batch. The chemostat then equilibrates to a new fixed point and the procedure is repeated until function and biomass reach stability. **A**: Time evolution of the relative abundances of individual strains for one realization of the system. Each colored line represents a different strain; strains appear and disappear as invasions and extinctions occur. **B**: Time evolution of functional occurrences $F_{i}$ for three different realizations of the system (distinguished by line style). Different colors represent different resources. 15 resources are given. The three realizations converge to the same functional profile despite starting from different initial conditions. **C**: Time evolution of the total biomass of the system. 20 realizations of the system are shown for each value of average resource income.

Starting from a clonal population, a diverse community is rapidly assembled. Strain abundances change abruptly following successful invasion events and continue changing over the whole duration of the simulations (Fig 2A).

However, the final community structure is remarkably simple if, instead of analyzing strain abundances, we focus on its functional composition. We define functional occurrence $F_{i}$ as the community consumption rate, averaged across individuals (see Materials and methods). After a short transient, the functional occurrences and the total biomass *N* relax to their respective stationary values $F_{i}^{*}$ and $N^{*}$, which are very reproducible across different realizations (Fig 2B and 2C).

This behavior reveals that two phases characterize the eco-evolutionary dynamics (see Fig 2). The first phase is an initial-condition-dependent transient, where the community structure is mainly shaped by rapid invasions. In the second phase, conversely, the community has converged to a stable functional composition, which we refer to as the “functional attractor” in the following, and slowly evolves while reaching the final strain-level equilibrium. It is important to note that this attractor represents a region in the functional space, rather than a single static point. Although the community’s functional profile remains globally stable and predictable, it can exhibit minor fluctuations as the underlying taxonomic composition shifts due to ongoing, albeit slow, evolutionary dynamics.

This result is stronger than the expectation that total resource consumption must balance supply at equilibrium. While the balance of consumption and supply constrains the total consumption rate of each resource, it does not, by itself, predict how resource consumption is distributed across individuals. The functional occurrence $F_{i}$ — the *fraction* of individuals consuming resource *i* — is uniquely determined by environmental parameters, independently of which specific strains is part of the community. This independence from the species pool is a non-trivial consequence of the metabolic trade-off, which allows the Lyapunov function governing the ecological dynamics to be expressed purely in terms of the total biomass *N* and the functional occurrences $\left\{F_{i}\right\}$, without reference to individual strain abundances (see Materials and methods). If the species pool is too limited to adequately explore the functional space, the community cannot converge to the predicted functional composition (Fig B in S1 Appendix).

Notably, the total biomass converges to a constant value during the second phase of the eco-evolutionary dynamics, which therefore affects only the relative abundance of strains. The sequence of invasions and extinctions of strains is determined by the interplay of fitness differences $\xi_{\mu }$ and niche differences, which are related to the dissimilarity of the resource preferences. Importantly, the trajectories of strain abundances are effectively constrained to occur within the lower-dimensional space determined by the constraints enforced through the functional occurrences $F_{i}^{*}$. The strains involved in this turnover typically differ in their resource preference vectors (i.e., they belong to different ecotypes), not just in their intrinsic fitness values. The functional redundancy is therefore genuine and not merely a consequence of near-identical strains replacing each other.

During this second phase, the community has thus reached “functional maturity”, and the subsequent evolution — driven by intrinsic fitness differences — only affects strain composition while leaving the functional composition unaltered.

### Infinite-pool model captures functional attractor properties

The stability and reproducibility of the functional attractor suggest that it is possible to predict analytically its properties. We considered a toy model of the eco-evolutionary dynamics that aims at mimicking the effective exploration of the phenotypic space performed by mutations. In particular, we consider only the ecological dynamics, initialized with an infinitely large species pool, which encompasses all possible strains (e.g., which would correspond to $2^{R}$ possible resource preferences in the case of $a_{\mu i}\in \{0,1\}$). A similar approach has been considered to study a simpler version of the model [31] (corresponding to the limit χ≫1 and no cross-feeding). The toy model further postulates a timescale separation between resource and population dynamics [31,32], which is not assumed in the full eco-evolutionary dynamics.

This simplified framework allows for analytical solutions. The consumer-resource-crossfeeding model with an infinite pool of diversity and no intrinsic fitness differences can be analytically solved. In the Materials and Methods, we show that the stationary functional occurrences $F_{i}^{*}$ and the total biomass $N^{*}$ are given by


$$\begin{array}{c} F_{i}^{*}=\min\left\{\frac{h_{i}^{eff}}{\chi }\frac{1}{N^{*}},1\right\} \end{array}$$ (3)

and


$$\begin{array}{c} N^{*}=\frac{\sum_{i}h_{i}^{eff}}{R(1+\chi \sum_{i}F_{i}^{*})}\;. \end{array}$$ (4)

Here, the parameter $h_{i}^{eff}$ is the effective resource inflow into the system, which accounts for both externally supplied resources and those internally recycled through cross-feeding. Specifically, $h_{i}^{eff}=(1-\ell )\sum_{j}B_{ij}h_{j}w_{j}$ where $B=(I-\ell D{)}^{-1}$ captures the amplification of resource availability due to metabolic recycling (see Materials and methods).

**Fig 3:**
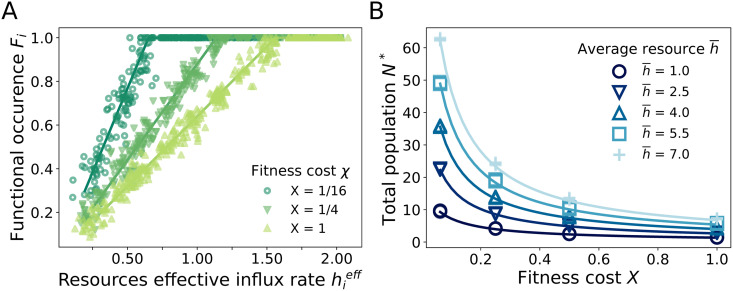
The theoretical predictions given by eqs. 14, 15 (solid lines) are reproduced by numerical integration of eqs. 5, 6 (markers). **A**: Occurrence of the phenotypes $F_{i}$ as a function of resource income rates $h_{i}$. According to equation 14 the most abundant resources (core) are consumed by all strains ($F_{i}$=1) while the remaining ones only by a fraction $F_{i}=\frac{h_{i}}{N^{*}\chi }$. Notice that increasing χ reflects in a decrease of the number of core resources. **B**: Dependence of the total equilibrium population $N^{*}$ (biomass) on the value of χ. Here we find a dependence on the average resource income $\overset{―}{h}$, which is absent for quantities in panel A. In each figure are represented 20 different noise realizations solutions of the system for each χ **(A)** and each $\overset{―}{h}$ **(B)**.

The analytical calculations are based on many simplifying assumptions (infinitely large pool of diversity, no explicit resource dynamics, absence of fitness differences) which do not strictly hold for the more complex setting of the eco-evolutionary model. The close agreement between these analytical predictions and the full simulation results suggests that the eco-evolutionary dynamics are effective at exploring the landscape of possible phenotypes, mimicking the ’infinite-pool’ assumption. Even with a finite set of evolving strains, the continuous introduction of mutations allows the community to eventually find and converge upon the same functionally optimal state predicted by the simplified model. This indicates that the properties of the attractor are primarily governed by fundamental environmental and metabolic constraints rather than the specific trajectory of evolution. Nevertheless, Fig 3 shows that the predictions of Eq. 3 and Eq. 4 accurately describe the outcomes of the eco-evolutionary dynamics.

Resources can be divided into two groups according to their effective influx rate $h_{i}^{eff}$. If the influx rate is larger than a critical value $h_{c}^{eff}$, then the ability to metabolize that resource is a “core” function, shared by all the individuals in the community (i.e., $F_{i}^{*}=1$ or equivalently $a_{\mu i}=1$ for all the strains present in the community). The value of $h_{c}^{eff}$ depends on both the spread of the effective influx rate (the variability among the $h_{i}$) and the metabolic cost χ. The higher the metabolic cost and the variability, the higher the critical influx rate threshold $h_{c}^{eff}$ and, consequently, the fewer the core resources.

At equilibrium, the resources with an influx rate below the critical threshold (i.e., the non-core resources) are consumed only by a fraction of the individuals. A linear relation links the functional occurrence $F_{i}^{*}$ with the effective resource influx rate $h_{i}^{eff}$. The slope of this relation is simply related to the metabolic cost and the total biomass, being equal to $(\chi N^{*}{)}^{-1}$ (see Materials and methods). By combining Eq. 3 and Eq. 4, one can obtain an explicit expression for $N^{*}$. Fig 2B shows that the analytical expression for $N^{*}$ (as a function of the metabolic cost χ) correctly matches the observations of the eco-evolutionary dynamics.

### Functional composition demonstrates robust redundancy against fitness variation

An emerging feature of the present framework is that the functional composition of communities is extremely robust to fitness differences. We further explore this aspect by considering the community response to variation in intrinsic fitness. This variation mimics the temporal or spatial heterogeneity of environmental factors that influence growth, such as abiotic factors (temperature, pH, salinity, etc.) or phages with different host ranges.

We consider two complementary scenarios, which aim at exploring cross-sectional (across communities) and longitudinal (over time) variation. In the cross-sectional case, we compare the eco-evolutionary outcomes of several communities that share the same resource input but have independent intrinsic fitness values. Under this scenario, two individuals with the same resource preference will have uncorrelated intrinsic fitnesses between two different communities.

The longitudinal case assumes instead that intrinsic fitness fluctuates over time according to an Ornstein-Uhlenbeck process with a characteristic autocorrelation timescale $\tau_{corr}$ (see Materials and methods). Over time ranges shorter than $\tau_{corr}$, intrinsic fitness is approximately constant. Over times larger than $\tau_{corr}$, intrinsic fitness decorrelates and becomes independent.

**Fig 4:**
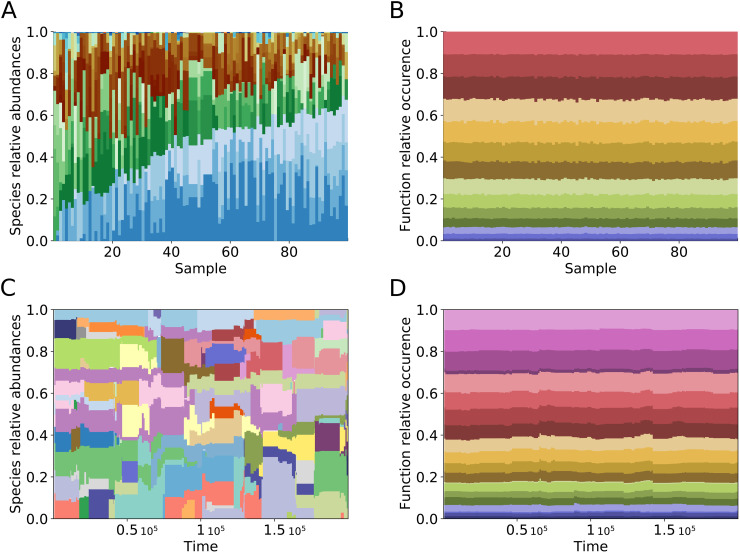
Fitness differences demonstrate functional stability. While strain composition becomes highly variable and heterogeneous, the functional composition is preserved and unaffected by intrinsic fitness variation. **A**, **B**: Cross-sectional comparison of equilibrium configurations across communities with different realizations of intrinsic fitness values (static, i.e., constant in time for each community). Each bar in A represents a different community; colors denote distinct strains. B shows the corresponding functional occurrences, which remain nearly identical across communities. **C**, **D**: Longitudinal dynamics of a single community where intrinsic fitness values fluctuate over time following an Ornstein-Uhlenbeck process (see Materials and methods). C shows that strain composition turns over continuously as fitness values change, while D shows that functional occurrences remain stable throughout.

Figure 4 shows the strain and functional composition of communities in the two scenarios described above. In the cross-sectional case (Fig 4A and 4B), each community is evolved to the functional attractor with independent intrinsic fitness values: the strain composition differs markedly across communities, while the functional profile is largely unaffected by fitness variation. The longitudinal case (Fig 4C and 4D) shows a single community evolving over time with fluctuating intrinsic fitness. Unlike Fig 2A, where the community is assembling from scratch (with both functional and taxonomic composition changing during the transient), Fig 4C depicts a community that has already reached the functional attractor. The subsequent taxonomic turnover is driven entirely by the fluctuating fitness landscape and proceeds at a rate controlled by the autocorrelation timescale $\tau_{corr}$ (Fig C in S1 Appendix). A linear time axis is used in Fig 4C (as opposed to the logarithmic axis in Fig 2) because the turnover dynamics are stationary, without the initial transient that requires log-scale visualization.

These observations clearly show that functional redundancy naturally emerges in complex consumer-resource-crossfeeding models, closely reproducing the phenomenology observed in microbial communities [12].

To assess the generality of our findings, we tested the robustness of our predictions beyond the assumption of universal cross-feeding. In the model, we initially assume that the cross-feeding matrix is universal (strain-independent). To test the robustness of our predictions to this assumption, we examined communities where each ecotype has its own randomized cross-feeding matrix (see Materials and methods). Even under these more realistic conditions, the functional occurrences $F_{i}^{*}$ still depend on the effective resource influx rates $h_{i}^{eff}$ in a manner consistent with our analytical predictions (Fig D in S1 Appendix). The relationship between functional composition and resource availability remains present, though with fluctuations around the ecotype-independent cross-feeding matrix case. Most importantly, functional composition remains robust to intrinsic fitness differences even when cross-feeding matrices vary across ecotypes, confirming that functional redundancy emerges even without universal metabolic stoichiometry.

### Robustness of the functional attractor

The functional attractor is robust across a wide range of model assumptions, but its existence requires specific conditions. We systematically explored the sensitivity of our results to key parameters and modeling choices.

*Conditions for the attractor*. The metabolic trade-off (χ>0) is essential: without it, the Lyapunov function no longer depends on the functional occurrences $\left\{F_{i}\right\}$, and no selection pressure favors a specific functional composition. Similarly, the species pool must be large enough, or the evolutionary dynamics must run long enough, for the community to effectively explore the functional space. When only a small number of strains is available and no evolutionary dynamics is allowed, the community cannot converge to the predicted functional composition (Fig B in S1 Appendix). The eco-evolutionary dynamics play a key role in mimicking the effect of an infinite species pool, allowing convergence even from a small initial community. The number of resources *R* does not qualitatively affect the existence of the attractor: the relationship between functional occurrence and effective resource influx holds for *R* ranging from 5 to 25 (Fig E in S1 Appendix).

*Fitness differences*. Small intrinsic fitness differences ($\epsilon \leq 10^{-2}$) do not alter the functional composition (Fig F in S1 Appendix). Larger differences (ϵ~0.1) partially disrupt the attractor, and very large differences (ϵ~0.6) destroy it entirely: the functional profile becomes decoupled from the resource input and dominated by the resource preferences of the fittest strains (Fig G in S1 Appendix). The relevant comparison is between ϵ and the metabolic cost χ: when ϵ/χ≫1, fitness effects dominate over metabolic constraints.

*Evolutionary parameters*. The mutation rate, the ratio of gene loss to gain events, and the relative contribution of horizontal gene transfer versus de novo mutation all affect the speed of convergence to the attractor but not its final state (Fig H in S1 Appendix).

*Functional response and trade-off shape*. The choice of functional response — linear ($r_{i}(c_{i})=c_{i}$) or Monod ($r_{i}(c_{i})=\mu_{max}c_{i}/(K_{s}+c_{i})$) — does not affect the functional composition (Fig I in S1 Appendix). Non-linear trade-offs, both super-linear (*H*(*z*) = (1 + *z*)^2^) and sub-linear (H(z)=log(1+z)), also produce convergence to a reproducible functional composition with core and non-core resources approximately linearly related to effective resource influx rates (Figs J and K in S1 Appendix).

*Cross-feeding structure*. Neither the strength nor the structure of the cross-feeding matrix affects convergence to the attractor (Fig L in S1 Appendix). The functional composition is robust even when cross-feeding matrices are ecotype-specific, varying from universal to fully strain-dependent stoichiometry (Fig D in S1 Appendix).

## Discussion

Our results shed light on the composition of large ecological communities. While previous work has demonstrated that consumer-resource models with fixed species pools and metabolic trade-offs converge to ecological equilibria with specific functional properties [31,32], our work extends this in three important directions. First, we show that explicit eco-evolutionary dynamics — where strains are not drawn from a fixed pool but arise through mutation and horizontal gene transfer — reproduce the same functional attractors, demonstrating that evolution effectively mimics an infinite species pool. Second, we incorporate cross-feeding, which introduces effective resource influx rates $h_{i}^{eff}$ that account for metabolic recycling, extending the analytical predictions to a wider class of models relevant to microbial communities [6]. Third, we show that the separation of the dynamics into a fast (functional) and a slow (taxonomic) phase provides a mechanistic explanation for the empirically observed phenomenon of functional redundancy [11,12], connecting theoretical ecology with metagenomics observations.

When the pool of diversity is not a-priori constrained but is instead allowed to evolve, the complex ecological dynamics can be decomposed in a fast, predictable, phase and a slow one, contingent on the (small, yet relevant) fitness differences. The community composition rapidly converges to a set of states, fully determined by resource availability. The ecological dynamics that follows is constrained on that subspace of solutions and is governed by the difference in relative fitness. Remarkably, this separation of fast and slow components directly maps into functional and taxonomic composition: the former is robust and governed only by effective resource influx rates, the latter is constrained by function, but free to move along functionally equivalent directions.

In our setting, the functional profile is defined by the average resource consumption rate. While it is expected that in an assembled community each niche is occupied (i.e., at least one ecotype is able to grow on each resource), our result is much stronger, showing that also the fraction of individuals able to grow on each resource is highly reproducible.

The functional robustness and functional redundancy are the direct consequences of the existence of the two dynamics phases that directly map onto taxonomic and functional variation. Functional robustness, the observation that functional composition is stable over time and across communities, originates from the existence of a functional attractor of the eco-evolutionary dynamics. While intrinsic fitness differences are small, they are not negligible as they determine the taxonomic composition within the functional attractor. Variation of the intrinsic fitness leads to functional redundancy, high taxonomic variability with conserved functional profile. The mechanism buffering the functional profile from this underlying taxonomic variation can be understood through a separation of selective pressures. The primary selective pressure, driven by resource availability and metabolic trade-offs, rapidly shapes the overall functional composition of the community, forcing it into the functional attractor. Once the community resides within this attractor, the direct selection pressure on function is weak. At this stage, the much smaller intrinsic fitness differences (determined by $\xi_{\mu }$) become the dominant factor in determining the competitive success among functionally similar strains. This secondary selection drives the turnover of strains without altering the conserved functional roles, as any new successful invader must still conform to the constraints imposed by the attractor.

The assumption of small intrinsic fitness differences is critical for observing functional robustness and redundancy. Increasing the magnitude of fitness differences also affects the functional profile. Typical intrinsic fitness differences of 1% do not substantially alter the functional composition (Fig F in S1 Appendix). However, larger differences (of the order of 10%) disrupt the structure of the functional attractor: the functional composition is determined by the resource preferences of the individuals with the largest intrinsic fitness, and the functional profile becomes largely decoupled from the resource input (see Figs F and G in S1 Appendix). Differences of 0.1% and smaller are indistinguishable from the analytical prediction, and the functional profile closely matches that predicted by the resource influx rates.

While our main analysis assumes universal cross-feeding stoichiometry across strains, this assumption is not critical to our main conclusions. The functional universality persists even when different ecotypes convert resources through distinct metabolic pathways with ecotype-specific cross-feeding matrices (Fig D in S1 Appendix). This robustness suggests that functional convergence arises from the constraints imposed by resource availability rather than from the specific details of metabolic conversion pathways. Even when individual ecotypes have unique metabolic stoichiometries, the community as a whole establishes an effective metabolic network. The functional attractor is then determined by the community-averaged effective resource influx ($h_{i}^{eff}$), which emerges from the collective metabolic activity of all coexisting strains, weighted by their abundances. This emergent, community-level property smooths over the underlying ecotype-specific variations, ensuring that the relationship between resource supply and functional composition remains predictable and robust.

The existence of these different regimes, where the functional composition is or is not affected by intrinsic fitness differences, is strictly related to the identification of the limiting factors shaping the communities. As mentioned previously, the intrinsic differences could be due to abiotic factors, but also to limiting factors other than resource availability (e.g., phages). If resources are limiting, we can expect that other factors will have a minimal effect on strain success. Conversely, if resources are not the limiting factors and other mechanisms determine strain growth and decline, the distribution of functional preferences in the population will not be robust, as it will be subject to the fluctuations of the other limiting factors.

Importantly, our results hold in the very specific setting of consumer-resource models considered here, where “function” is interpreted as the ability of an individual to grow on a given substrate. Our framework could be extended to explicitly include the factors responsible for intrinsic fitness differences (e.g., temperature ranges).

The metabolic trade-off is an essential ingredient of our framework. We focused on a fitness cost that is linear in the total rate of resource consumption, generalizing models with a fixed total rate [31,32] by including a basal maintenance cost. This basal cost becomes negligible if the cost per gene, relative to the basal cost, becomes very large. The presence of a non-zero basal cost determines the existence of core resources, whose consumption is shared by all individuals in the community. The form of the functional attractor is a mathematical consequence of the linearity of the metabolic trade-off. For linear trade-offs, the functional attractor is fully specified by the functional composition and is, in the limit of negligible fitness differences, independent of how functions are distributed across ecotypes. While non-linear trade-offs [33] could, in principle, affect the properties of the eco-evolutionary attractor, we explicitly consider both super-linear and sub-linear trade-offs and show that our results are qualitatively unchanged. The taxonomic composition is largely affected by fitness differences, while the functional composition remains robust. The stable functional composition displays core and non-core resources, which are (at least approximately) linearly related to the effective resource influx rates.

A remarkable aspect of our framework is that functional composition — as opposed to taxonomic composition — naturally emerges as the relevant, reproducible degree of freedom well suited for characterizing ecological communities. Our results demonstrate that the emergence of a stable and reproducible functional composition is a universal feature of consumer-resource-crossfeeding models [6]. This property is likely to hold more generally and not be restricted to consumer-resource systems or microbial communities. We expect that a similar approach could be developed to study mutualistic communities or pathogen dynamics.

## Materials and methods

### Definition of the model

We consider a consumer-resource model in presence of cross-feeding [17], which describes the dynamics of population abundances $n_{\sigma }$ (for σ∈𝒮) and resources concentration $c_{i}$ (for i∈R). Changes in population abundance are defined by


$$\begin{array}{c} \frac{dn_{\sigma }}{dt}=n_{\sigma }\left(\eta_{\sigma }\sum_{i\in R}{ℰ}_{i\sigma }^{g}-\delta_{\sigma }\right)\;. \end{array}$$ (5)

where $\delta_{\sigma }$ is a death term and $\eta_{\sigma }$ is the efficiency of the conversion of energy into biomass. ${ℰ}_{i\sigma }^{g}$ is the energy flux used for strain σ to grow from metabolite *i*. We can similarly define the energy flux ${ℰ}_{i\sigma }^{in}$ into a cell from resource *i* and the energy released in the environment by the cell ${ℰ}_{i\sigma }^{out}$ in the form of other metabolites obtained from from metabolites of type *i*. The associated dynamics of resource concentration $c_{i}$ is defined by


$$\begin{array}{c} \frac{dc_{i}}{dt}=h_{i}(c_{i})-\frac{1}{w_{i}}\sum_{\sigma \in 𝒮}n_{\sigma }{ℰ}_{i\sigma }^{in}+\frac{1}{w_{i}}\sum_{\sigma \in 𝒮}n_{\sigma }{ℰ}_{i\sigma }^{out}\;, \end{array}$$ (6)

where $w_{i}$ defines the conversion between energy and concentration of resource *i*. The function $h_{i}(c_{i})$ specifies the dynamics of resource concentration in absence of consumers.

We assume that energy fluxes used for growth are a constant fraction 1−ℓ of the total ones: ${ℰ}_{i\sigma }^{g}=(1-\ell ){ℰ}_{i\sigma }^{in}$. The energy fluxed from secreted metabolites is then given by ${ℰ}_{i\sigma }^{out}=\ell \sum_{j\in R}D_{ij}{ℰ}_{j\sigma }^{in}$. The cross-feeding matrix element $D_{ij}$ defines energy conversion between resource *j* and resource *i*. Energy conservation implies $\sum_{i}D_{ij}=1$.

The energy flux ${ℰ}_{i\sigma }^{in}$ takes the form


$$\begin{array}{c} {ℰ}_{i\sigma }^{in}=w_{i}\nu_{i}a_{\sigma i}r_{i}(c_{i})\;, \end{array}$$ (7)

where $r_{i}(c_{i})$ is a non-decreasing function of the concentration of resource *i* and $\nu_{i}$ is the maximal intake rate of resource *i*. The elements $a_{\sigma i}\in [0,1]$ measure the intake rate of metabolite *i* strain σ relative to the maximum $\nu_{i}$. Here we focus on the case of externally supplied resources $h_{i}(c_{i})=h_{i}$, which assumes that dilution is negligible when the total population is around the carrying capacity [32].

We focus on a linear metabolic trade-off by assuming for death rates and yield the expression


$$\begin{array}{c} \frac{\delta_{\sigma }}{\eta_{\sigma }}=\frac{1}{\tau }\left(1+\chi \sum_{j\in R}a_{\sigma j}\right)(1-\xi_{\sigma })\;. \end{array}$$ (8)

Without loss of generality, we can set the timescale τ=1 equal for all strains, as differences in τ can be reabsorbed in the definition of $\xi_{\sigma }$. In the simple setting of $a_{\sigma j}\in \{0,1\}$, the parameter χ measures the cost of being able to metabolize each metabolite (χR is the fitness metabolic cost of a generalist).

### Functional attractor

In the eco-evolutionary simulations, we always consider resources and populations changing over a similar timescale. To make analytical progress we approximate the full dynamics with the effective one obtained by assuming timescale separation — i.e., resource concentrations equilibrate faster than the changes in population abundances. We underline that we assume the separation of timescales only as an approximation, for the purpose of predicting analytically the outcomes of the numerical simulations, which are always obtained with explicit resource dynamics.

In this case, one can effectively describe the dynamics of populations as


$$\begin{array}{c} \frac{dn_{\sigma }}{dt}=n_{\sigma }\left(\eta_{\sigma }\sum_{i\in R}a_{\sigma i}\nu_{i}\frac{h_{i}^{eff}}{\sum_{\mu \in 𝒮}n_{\mu }a_{\mu i}\nu_{i}}-\delta_{\sigma }\right)\;, \end{array}$$ (9)

where $h_{i}^{eff}=(1-\ell )\sum_{j\in R}B_{ij}h_{j}w_{j}$ and the matrix $B=(I-\ell D{)}^{-1}$. It is useful to notice that, in the limit χ→∞ and $h_{i}^{eff}\to \infty$ (such that the ration $h_{i}^{eff}/\chi$ is finite in the limit) reduces to the model with constant total energy budget [31,32]. It is known [31] that


$$\begin{array}{c} L(\{n\})=\sum_{\sigma }\frac{\delta_{\sigma }}{\eta_{\sigma }}n_{\sigma }-\sum_{i}h_{i}^{eff}\log\left(\sum_{\sigma }\nu_{i}a_{\sigma i}n_{\sigma }\right)\;, \end{array}$$ (10)

is a Lyapunov function. With our choice for the metabolic trade-off (8), such functional can be conveniently rewritten as


$$\begin{array}{c} L(\{n\})=\sum_{\sigma }n_{\alpha }\left(1+\chi \sum_{j\in R}a_{\sigma j}\right)(1-\xi_{\sigma })-\sum_{i}h_{i}^{eff}\log\left(\sum_{\sigma }\nu_{i}a_{\sigma i}n_{\sigma }\right)\;. \end{array}$$ (11)

We then introduce the total population size $N=\sum_{\sigma \in 𝒮}n_{\sigma }$ and define the functional abundances $F_{i}$ as


$$\begin{array}{c} F_{i}=\sum_{\sigma \in 𝒮}a_{\sigma i}\frac{n_{\sigma }}{N}\;, \end{array}$$ (12)

which correspond to the fraction of individuals that are able to metabolize resource *i*. Interestingly, and surprisingly, when $\xi_{\sigma }=0$, the Lyapunov function can then be written as function of *N* and {*F*} alone:


$$\begin{array}{c} L(N,\{F\})=N\left(1+\chi \sum_{j\in R}F_{j}\right)-\sum_{j\in R}h_{j}^{eff}\log\left(N\nu_{j}F_{j}\right)\;. \end{array}$$ (13)

The fact that the Lyapunov function depends only on the total biomass and the functional profile already suggest, even if it does not imply, that functional abundances are the relevant variable for the study of community composition.

By minimizing the function over $F_{i}$ in [0,1] one obtains


$$\begin{array}{c} F_{i}^{*}=\min\{1,\frac{1}{N^{*}}\frac{h_{i}^{eff}}{\chi }\}\;, \end{array}$$ (14)

where the total biomass is the solution of


$$\begin{array}{c} N^{*}=\frac{\sum_{j\in R}h_{j}^{eff}}{\left(1+\chi \sum_{j\in R}F_{j}^{*}\right)}\;. \end{array}$$ (15)

These equations can be solve iteratively, starting from $F_{i}=1$ ∀i and $N=\sum_{j\in R}h_{j}^{eff}/(1+\chi R)$.

In the case with no intrinsic fitness differences ($\xi_{\sigma }=0$), the equilibrium solutions are identified by equations 14 and 15. For a given system, a fraction of resources will be core resources, i.e., shared by everyone $F_{i}^{*}=1$. These core resources are the ones for which $h_{i}^{eff}\geq \chi N^{*}$.

### Eco-evolutionary dynamics

The mutation probability of a preference of resource *i* in strain μ depends on whether μ consumes or not *i*. The rate *U*_-,*i*_ at which a mutant $\tilde{\mu }$ stops consuming resource *i* (the parent has $a_{i}=1$ and the mutant $a_{i}=0$) is constant, independent of *i*, and equal to *U*_-_. The rate at which a mutant starts consuming a resource *i* (the parent has $a_{i}=0$ and the mutant $a_{i}=1$) equals to $U_{+,i}=U_{+}(P_{h}F_{i}+P_{dn})$. The quantity $P_{h}$ is the probability that an addition happens because of horizontal gene transfer, while $P_{dn}=1-P_{h}$ the probability of “de-novo” mutations. The rate of horizontal transfer is proportional to the frequency $F_{i}$ of that allele in the population, while the rate of a de-novo mutation is independent of *i*.

The rate at which the resource preference *i* mutates in strain μ is then equal to


$$\begin{array}{c} W_{\mu ,i}^{mut}=b_{\mu }n_{\mu }\left(a_{\mu i}U_{-,i}+(1-a_{\mu i})U_{+,i}\right)\;, \end{array}$$ (16)

where $b_{\mu }$ is the per-capita birth rate on strain μ, which is equal to


$$\begin{array}{c} b_{\mu }=\eta_{\mu }\sum_{j}a_{\mu j}r_{j}(c_{j})\;. \end{array}$$ (17)

In theory one could expect a new mutant to have abundance 1. The initial phase of its dynamics is then dominated by demographic stochasticity, with many mutants going to extinction despite having a positive (average) growth rate. In our framework, we do not consider this effect of demographic stochasticity explicitly, but we include it effectively. Since the initial abundance of the mutant $\tilde{\mu }$ is a small fraction of the total population, its stochastic dynamics can be approximated by a stochastic exponential growth. In this regime, the per-capita birth rate of the mutant is given by $\left(1-\ell )\sum_{i}a_{\tilde{\mu }i}r_{i}(c_{i}^{*}\right)$, where $c_{i}^{*}$ is the concentration of resource *i* prior to the mutant arrival. The per-capita death rate of the mutant reads $\left(1+\chi \sum_{i}a_{\tilde{\mu }i})(1-\xi_{\tilde{\mu }}\right)$. Under the assumption of a stochastic exponential growth the survival probability is given by


$$\begin{array}{c} p_{\tilde{\mu }}^{surv}=1-\min\left(1,\frac{\left(1+\chi \sum_{i}a_{\tilde{\mu }i})(1-\xi_{\tilde{\mu }}\right)}{\left(1-l)\sum_{i}a_{\tilde{\mu }i}r_{i}(c_{i}\right)}\right)\;. \end{array}$$ (18)

The strain intrinsic fitness values $\xi_{\mu }$ are independently drawn from a Gaussian distribution with mean 0 and standard deviation ϵ.

By calculating all these quantities for all possible mutations of all existing strains, one obtain the rate of invasion $W_{i\mu }^{inv}$ of a mutant $\tilde{\mu }$ which is obtained by changing the resource preference of strain μ for resource *i*. The rate of invasion $W_{i\mu }^{inv}$ reads


$$\begin{array}{c} W_{\mu ,i}^{inv}=W_{\mu ,i}^{mut}p_{\tilde{\mu }}^{surv}\;, \end{array}$$ (19)

where the mutant $\tilde{\mu }$ differ from μ in the resource preference *i*.

We simulate the eco-evolutionary dynamics as a sequence of discrete small time steps Δt. After a step of integration of equations 5 and 6 we update the values of $W_{i\mu }^{inv}$, as they depend on strain abundances, and checked whether a mutant appeared. Each mutant, identified by the parent strain μ and a resource *i*, has probability $W_{i\mu }^{inv}\Delta t$ to invade. If such an event occurs, the new mutant is introduced with an initial relative density equal to 10^-5^.

If no mutations appear for a long enough time, the ecological dynamics (obtained by integrating equations 5 and 6) reach an equilibrium point, identified numerically when the absolute value of the population growth rate is lower than 10^-4^. If the strain abundances are not changing, also the rates of invasions $W_{i\mu }^{inv}$ are constant in time (until the next successful invasion), and one can use a Gillespie algorithm. The time of the next successful invasion is drawn from an exponential distribution with average $T=1/\sum_{i\mu }W_{i\mu }^{inv}$. The probability that the new mutant will replace strain μ differing in resource preference *i* is simply $TW_{i\mu }$.

### Choice of parameters and sensitivity analysis

The results presented in this paper were achieved using generic parameters, whose details can affect the distribution of taxa or relaxation time but not the macroscopic observables that characterize the functional attractor. In order to quantify the convergence to the functional attractor, we measure the discrepancy between the functional composition of the community during its eco-evolutionary trajectory and the functional composition predicted by equations 14 and 15. As a measure of the discrepancy, we consider the Kullback-Leibler divergence between the normalized functional profiles


$$\begin{array}{c} d_{KL}=\sum_{i}\frac{F_{i}^{*}}{\sum_{j}F_{j}^{*}}\log\left(\frac{F_{i}^{*}}{\sum_{j}F_{j}^{*}}\frac{\sum_{j}F_{j}}{F_{i}}\right)\;. \end{array}$$ (20)

The divergence $d_{KL}$ is equal to zero if and only if the functional composition of the community (quantified by the $F_{i}$) matches the analytical expectation, i.e., if $F_{i}=F_{i}^{*}$ for all the *i*.

We considered $\eta_{\sigma }=\nu_{i}=w_{i}=1$ in eq. 5 and 6. These choices do not affect the results, as they do not affect the ecological fixed point and its stability property (up to a rescaling of the abundances and concentrations). The timescale τ was also set to 1, without loss of generality.

Functional responses. In the main text we considered $r_{i}(c_{i})=c_{i}$. We explore the effect of non-linear intake functions $r_{i}(c)$ by considering a Monod-like form $r_{i}(c_{i})=\mu_{max}c_{i}/(c_{i}+K_{s})$ with different values of $K_{s}$. Fig I in S1 Appendix shows that the value of $K_{s}$ has no effect on the functional composition of evolved communities.

Intrinsic fitness differences. The intrinsic fitness of any new mutant was drawn from a Gaussian distribution with mean zero and variance $\epsilon^{2}$, independently of the fitness of the parent. In the main text we considered ϵ=0.001. Fig F in S1 Appendix explores the sensitivity of the results to the magnitude of the noise. Much larger values of noise (of the order 0.1) often disrupt the properties of the manifold. For instance, strains not consuming core resources are still able to survive because of high intrinsic fitness. For intrinsic fitness differences with a width of the order of 10^-2^, the functional composition converges to the analytical prediction, which becomes more and more accurate as fitness differences decrease.

Longitudinal fitness fluctuations. In the longitudinal scenario (Fig 4C and 4D), the intrinsic fitness of each existing strain fluctuates over time according to an Ornstein-Uhlenbeck process:


$$\begin{array}{c} \frac{d\xi_{\mu }}{dt}=-\frac{\xi_{\mu }}{\tau_{corr}}+\epsilon \sqrt{\frac{2}{\tau_{corr}}}\;\zeta_{\mu }(t)\;, \end{array}$$ (21)

where $\tau_{corr}$ is the autocorrelation timescale and ϵ controls the stationary standard deviation of the fitness fluctuations. The term $\zeta_{\mu }(t)$ is a delta-correlated noise. This process ensures that, at stationarity, the intrinsic fitness of each strain is drawn from a Gaussian distribution with variance $\epsilon^{2}$, matching the cross-sectional case. The autocorrelation timescale $\tau_{corr}$ determines the rate at which the taxonomic composition turns over: faster decorrelation leads to more rapid strain replacement. Fig C in S1 Appendix quantifies the autocorrelation of taxonomic and functional composition for different values of $\tau_{corr}$, confirming that functional composition remains stable regardless of the turnover rate.

Intrinsic fitness staircase evolution. To ensure that our specific choice of intrinsic fitness evolution was not key to our results we repeated our simulations using a staircase model for the evolution, where the intrinsic fitness of the offspring is inherited from the parent with a multiplicative mutation $\xi_{parent}\to \xi_{offs.}=\xi_{parent}e^{\epsilon {\tilde{\xi }}_{\mu }}$. To ensure that this would not bring the system towards diminishing intrinsic fitness differences we rewrote the death rate as


$$\begin{array}{c} \left(1+\chi \sum_{i}a_{\mu i}\right)\xi_{\mu } \end{array}$$

Such a model returned the same results as the main model used in the manuscript as shown in Fig A in S1 Appendix.

Timescale interpretation. Time in the model is measured in units of the inverse dilution rate τ. In chemostat-like microbial systems, dilution rates are typically D~0.1−1 per hour, so one model time unit corresponds to approximately 1−10 hours. Convergence to the functional attractor occurs within $\sim 10^{7}$ time units (corresponding to $\sim 10^{3}-10^{4}$ years), after which the eco-evolutionary dynamics enters the second, taxonomic phase. The convergence timescale is controlled by the mutation rate: higher mutation rates accelerate convergence (Fig H in S1 Appendix). The key result, however, is the existence and universality of the functional attractor, not the precise convergence time.

Structure of the cross-feeding matrix. The strength of cross-feeding ℓ has no effect on convergence to the functional attractor (Fig L in S1 Appendix). The cross-feeding matrix *D* has been chosen following ref. [34]. The entries were extracted according to a Dirichlet distribution, where the resources are in three classes. We considered an effective sparsity of *s* = 0.1. The fraction of resources remaining in the same class was $f_{s}=0.7$ while the ones going to the waste class is $f_{w}=0.28$. The structure of the cross-feeding matrix *D* does not affect the stationary functional composition of the community. Fig L in S1 Appendix the structure described above with one obtained by sampling all the entries from a uniform distribution (which correspond to the single class case and no-sparsity of ref. [20], observing no difference in the results).

Mutation rates. Fig H in S1 Appendix shows that the outcomes of the evolutionary trajectories are independent of frequencies of the different mutation steps. We varied the (average) total mutation rate $U_{tot}=(U_{+}+U_{-})/2$, the ratio $U_{-}/U_{+}$ between mutation leading to deletions of resource preferences (with rate *U*_-_) and the ones leading to additions (*U*_+_), and the ratio $P_{h}/P_{dn}$ between horizontal gene transfer and de-novo mutations. While the total mutation rate, and partially the ratio $U_{-}/U_{+}$, affected the evolutionary trajectories and speed of adaptation, none of these parameters affected the convergence of the functional composition to the predicted attractor.

Non-linear tradeoff. Both the eco-evolutionary simulations and the analytical approximation are based on the assumption that the metabolic cost is linear in the consumed resources, as expressed in equation 8. In general, one could assume a non-linear tradeoff [33] that takes the form


$$\begin{array}{c} \frac{\delta_{\sigma }}{\eta_{\sigma }}=\frac{1}{\tau }H\left(\chi \sum_{j\in R}a_{\sigma j}\right)(1-\xi_{\sigma })\;, \end{array}$$ (22)

where *H*(*z*) is an arbitrary non-linear, monotonically increasing, function. The linear case, on which we focus in the main text, corresponds to *H*(*z*)=1 + *z*. We considered the outcomes of the evolutionary trajectories in the case of a super-linear cost (*H*(*z*)=(1 + *z*)^2^, in Fig J in S1 Appendix) and a sub-linear cost (H(z)=log(1+z), in Fig K in S1 Appendix). In both scenarios, the functional composition converges to reproducible values, minimally affected by fitness differences. On the other hand, the taxonomic composition is much largely affected by fitness differences. Similarly to the linear metabolic cost functions, some resources correspond to core-functions ($F_{i}^{*}=1$) while the functional occurrences $F_{i}^{*}$ of non-core resources are linearly related to the effective influx rates $h_{i}^{eff}$. These evolutionary outcomes, obtained under non-linear metabolic costs, confirm the generality of our results beyond the linear metabolic cost case.

Ecotype-specific cross-feeding matrix. One important assumption of our framework is that the cross-feeding matrix is ecotype-independent, assuming a “universal stoichiometry”. In order to text the generality of our results we studied the effect of species specific-matrices, with variable degrees of universality.

We generate a matrix common to all species ${\tilde{D}}_{ij}$ and one characterizing the ecotype-specific component $B_{ij}^{\mu }$. These matrices were independently generated as described above. The cross-feeding matrix of each species was defined as


$$\begin{array}{c} D_{ij}^{\mu }=(1-\lambda ){\tilde{D}}_{ij}+\lambda B_{ij}^{\mu }\;, \end{array}$$ (23)

where λ quantifies the degree of ecotype-specifity. The case considered in the main text (universal stoichiometry) corresponds to λ=0, while the full ecotype-specific case corresponds to λ=1.

Fig D in S1 Appendix shows the robustness of our results for different values of ecotype-specificity (λ∈[0,1]). While increasing ecotype-specificity produces larger departures from the exact solution of universal stochiometry (λ=0), the observed functional composition is still similar to what predicted by the average cross-feeding matrix. This observation is even stronger if the averaging is weighted by strain population abundances (panel B of Fig D in S1 Appendix).

## Supporting information

S1 Appendix Supplementary figures. Supporting figures (Fig A–Fig L), together with their legends, supporting the results and the robustness and sensitivity analyses presented in the main text. The figures are ordered by their first citation in the text.
(PDF)
