# Supplementary material for: Eco-evolutionary dynamics lead to functionally robust and redundant communities
Source: PLoS Comput Biol. 2026 Jul 21;22(7):e1014437. doi: 10.1371/journal.pcbi.1014437 (PMC13432115; doi:10.1371/journal.pcbi.1014437)
Supplement: S1 Appendix — Supporting figures (Fig A–Fig L), together with their legends, supporting the results and the robustness and sensitivity analyses presented in the main text. The figures are ordered by their first citation in the text. (PDF) [file pcbi.1014437.s001.pdf]

S1 Appendix

Supplementary figures

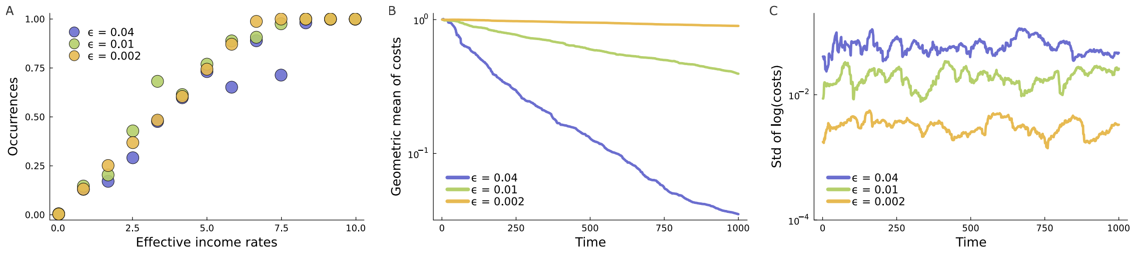

Fig A. The community reaches the functional attractor in a setting where the intrinsic fitness ξ_μ evolves in a staircase model. A: the relationship between functional occurrence F_i and effective resource influx h_i^{eff} holds across different mutation amplitudes ϵ. B: the average intrinsic fitness cost decreases in time while the standard deviation of the intrinsic-fitness logarithms is kept constant (C). This highlights that, while the mean value decreases, the variability of the costs is kept constant.

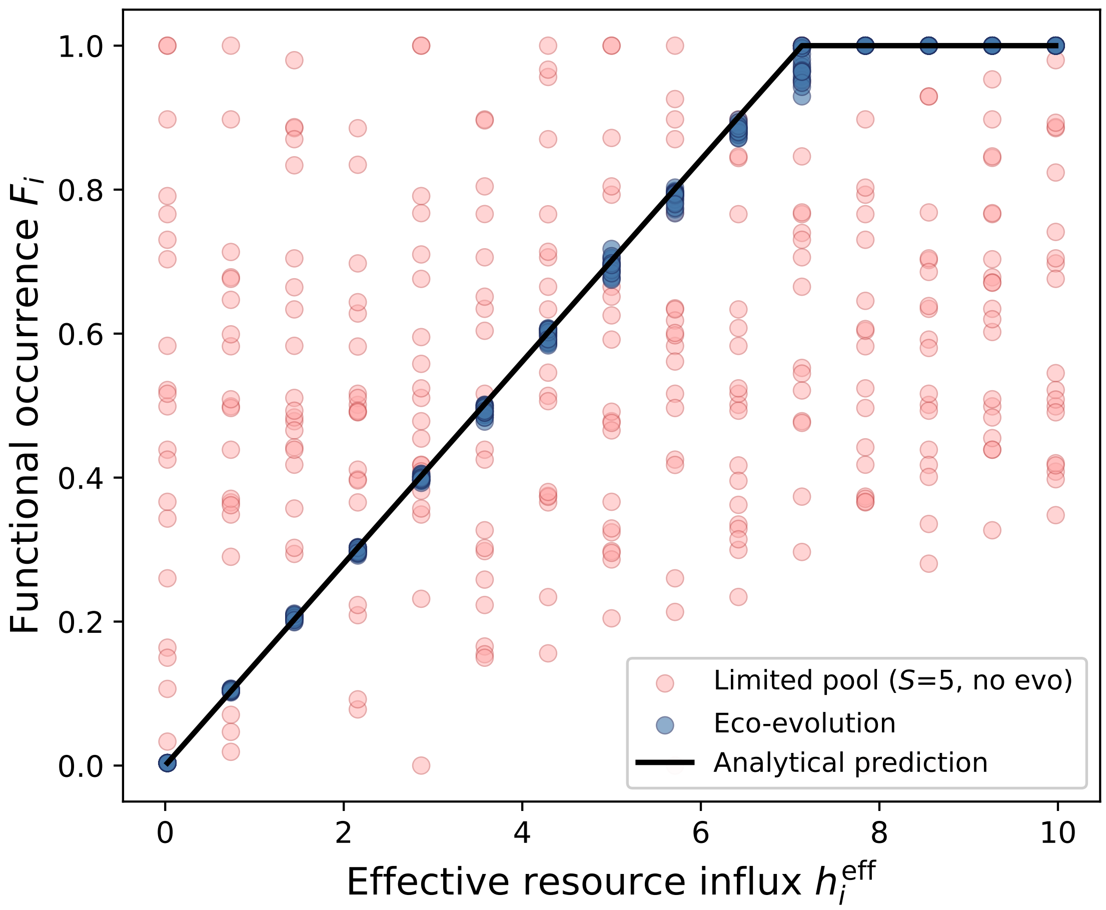

Fig B. A limited species pool prevents convergence to the functional attractor. With a limited species pool (5 strains, 15 resources, no evolution), the community cannot converge to the predicted functional attractor because the available diversity is insufficient to explore the functional space (red). In contrast, eco-evolutionary dynamics starting from the same small number of strains successfully converge to the analytical prediction (blue, solid line), demonstrating the essential role of evolution in mimicking an infinite species pool.

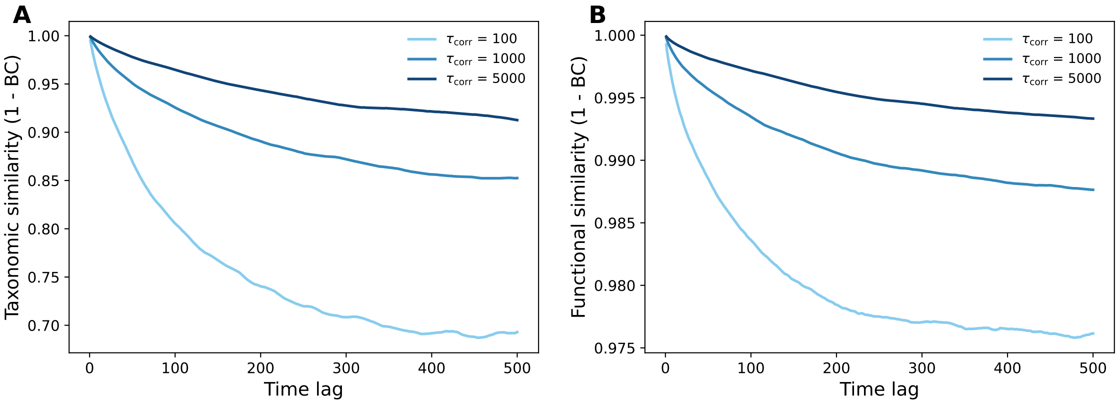

Fig C. Autocorrelation of taxonomic and functional composition under fluctuating intrinsic fitness. A community pre-evolved to the functional attractor is subjected to intrinsic-fitness fluctuations modeled as Ornstein–Uhlenbeck processes with different autocorrelation timescales τ_{corr}. A: taxonomic similarity (measured as $1-$ Bray–Curtis dissimilarity) as a function of time lag. Faster fitness fluctuations (smaller τ_{corr}) lead to faster decorrelation of strain composition. B: functional similarity as a function of time lag. Regardless of the fitness autocorrelation timescale, the functional composition remains nearly perfectly autocorrelated, confirming the robustness of the functional attractor to ongoing taxonomic turnover.

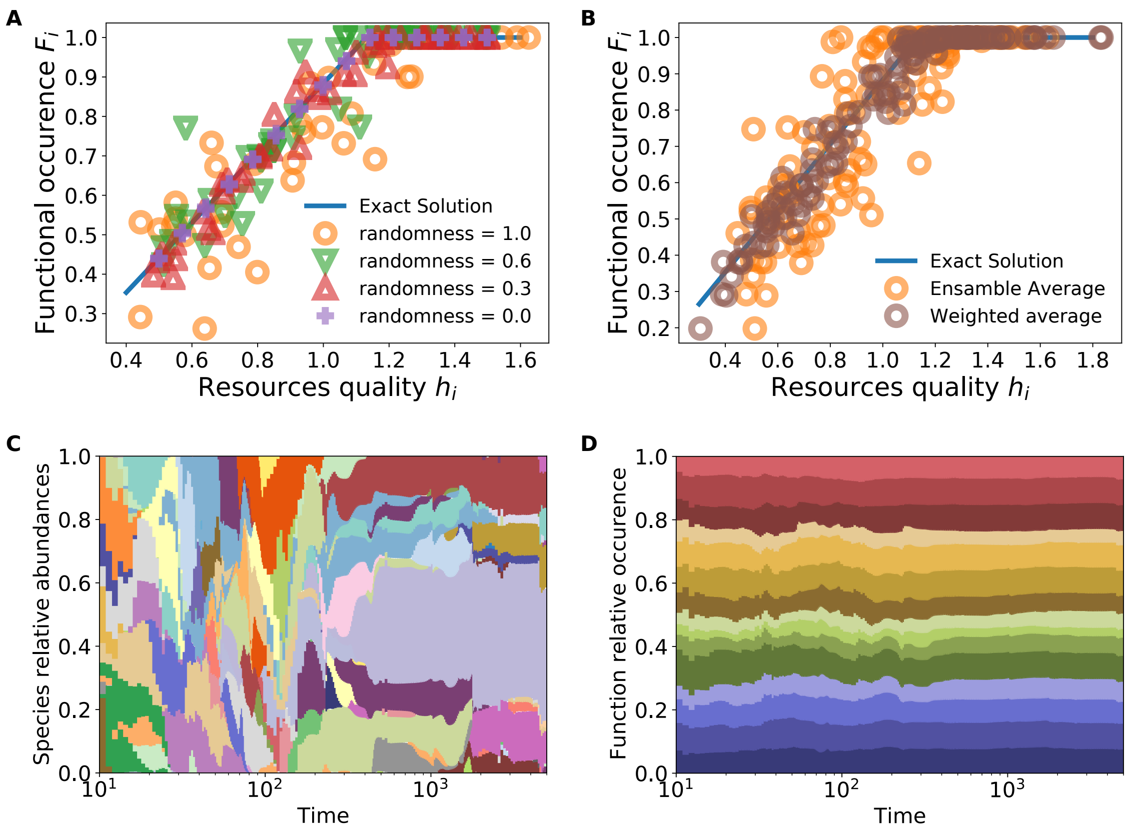

Fig D. Evolutionary outcomes under ecotype-specific cross-feeding matrices. All panels consider populations with an ecotype-specific cross-feeding matrix. The ecotype-specific matrix is generated by combining two matrices, as described in the main text. Panel A shows that the functional occurrences F_i^* depend on the effective resource influx rates h_i^{eff} in a similar fashion to what is observed for the case with a species-independent cross-feeding matrix, for different degrees of ecotype-specificity (as quantified by λ). The effective resource quality h_i^{eff} is calculated using the cross-feeding matrix averaged across strains. Panel B compares the results obtained by considering the average cross-feeding matrix with the ones obtained by weighting the averaging of the cross-feeding matrix by the population abundance of the strain. The other panels show the taxonomic (panel C) and functional (panel D) composition of different communities evolved in independent environments, characterized by the same effective resource influx rate h_i^{eff} but different intrinsic fitness values ξ_σ.

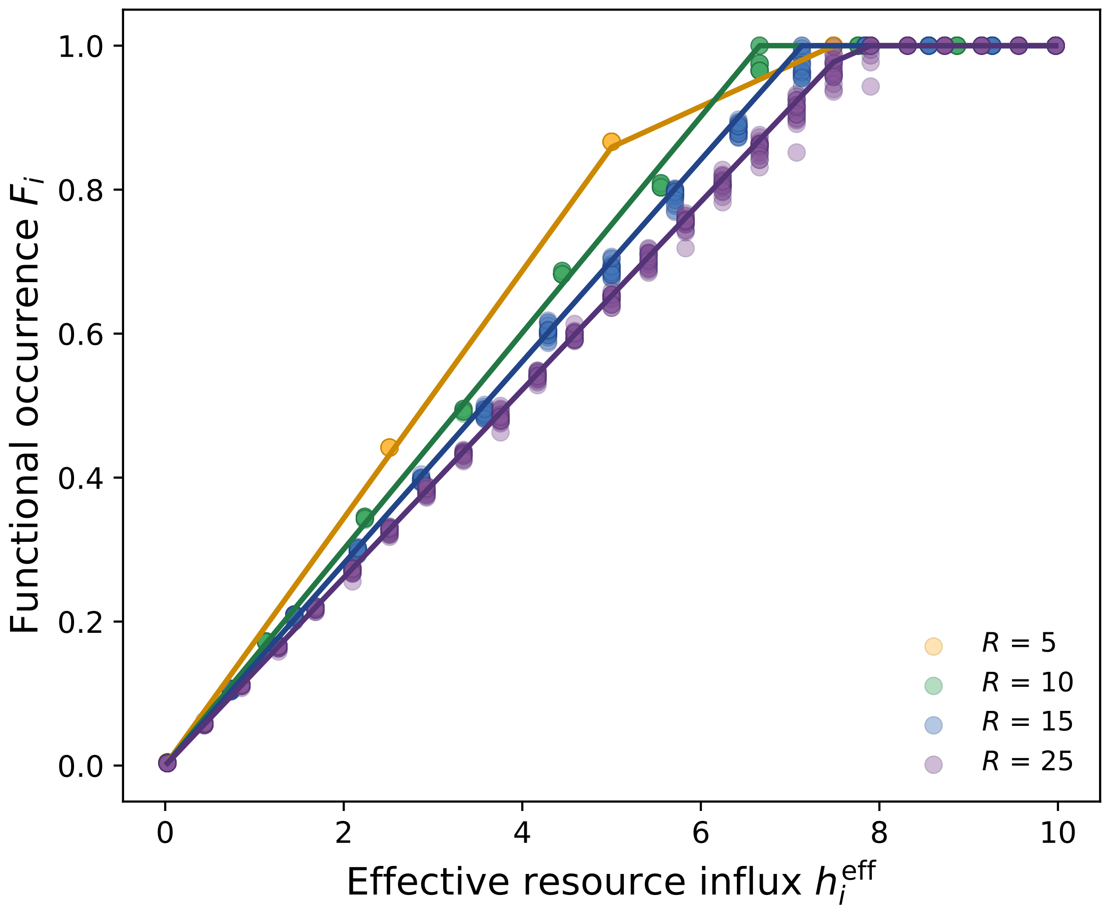

Fig E. Functional attractor for different numbers of resources R. The relationship between functional occurrence F_i and effective resource influx h_i^{eff} holds across different numbers of resources ($R = 5, 10, 15, 25$). Markers show the results of 20 realizations for each R; solid lines show the corresponding analytical predictions of the main text. The number of resources is representative of the diversity of carbon sources or nutrients available in microbial environments.

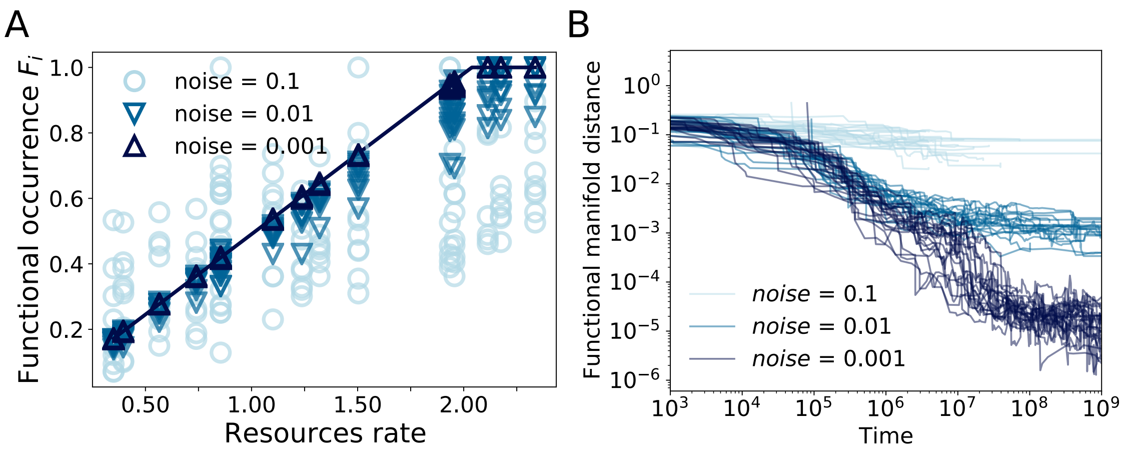

Fig F. Noise amplitude on fitness affects the convergence to the functional manifold. 20 realizations for three different amplitudes, $\epsilon = 10^{-3}$ (dark blue), $\epsilon = 10^{-2}$ (blue) and $\epsilon = 10^{-1}$ (light blue). A: final functional occurrences of the samples. In the case of $\epsilon = 0.1$ the results fall very far from the noiseless theoretical predictions. B: distance from the manifold as a function of time. The distance d_{KL} is defined in the main text. In all simulations all the other parameters were set to the same values used in the main text.

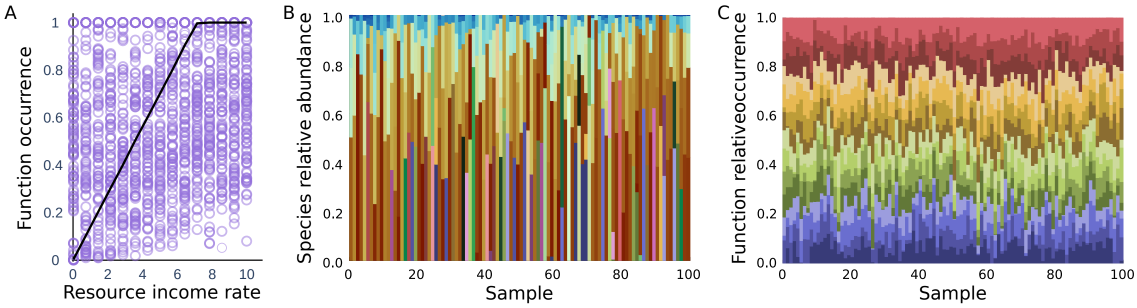

Fig G. Large noise amplitude destroys the functional attractor. The panels show 100 realizations for $\epsilon = 0.6$, where intrinsic fitness dominates over resource strategies. Panel A shows the lack of a relation between functional occurrence and the resource income rate. As a consequence, the high taxonomic variability due to the variation in intrinsic fitness (panel B) corresponds to a large variability of the functional composition (panel C).

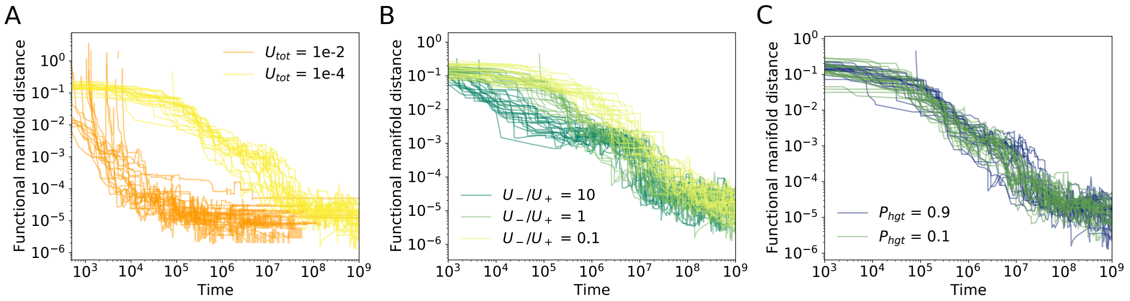

Fig H. The choice of evolutionary parameters does not affect the distance from the functional manifold but can modify the path walked to reach it. 20 realizations for every choice of the parameters are shown. A: effects of the mutation rate U_{tot}. Two values are considered, a fast mutation rate ($U_{tot} = 10^{-2}$) in orange and a slow one ($U_{tot} = 10^{-4}$) in yellow. B: effects of the ratio between function-loss and function-gain rates. Dark green for the case where losing a gene is more probable than gaining it, light green for the even case, and yellow for the samples where losing a function is less likely than gaining it. C: influence of the probability of gaining new genes via horizontal gene transfer (P_{hgt}) versus spontaneous mutation. The distance is defined in the main text. In all simulations all the other parameters were set to the same values used in the main text.

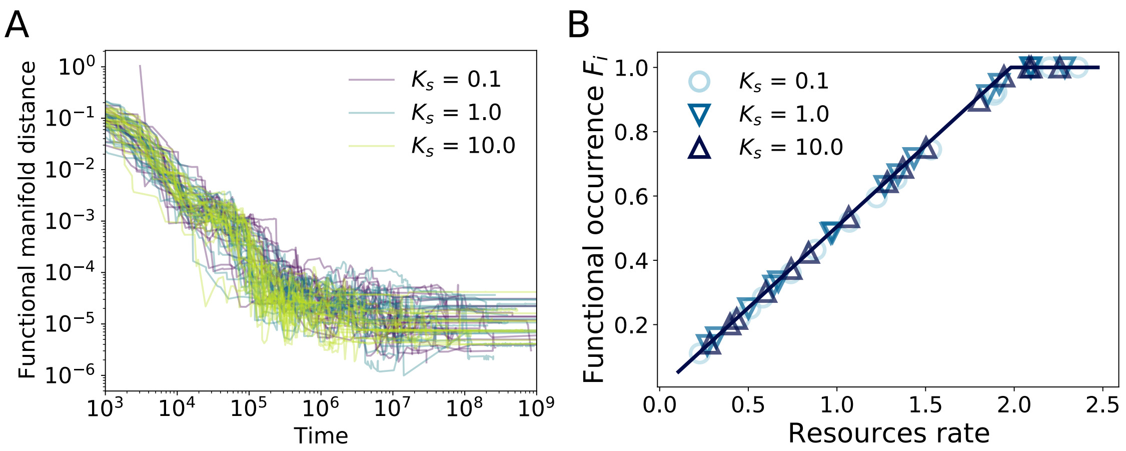

Fig I. The properties of the manifold are insensitive to the choice of the intake function $r_i(c_i)$. Both the linear response ($r_i(c_i) = c_i$) and the Monod response ($r_i(c_i) = \mu_{max}\, c_i/(K_s + c_i)$) reach the manifold with the same behavior. In A we show the time evolution of the distance from the theoretical manifold for 20 trajectories for every choice of K_s, and in B the functional occurrence for one realization for each K_s. This parameter is not determinant in the behavior of the convergence to the manifold. The constant $\mu_{max} = (2 + K_s)(2 + \chi)$ is chosen to ensure that the growth rate is higher than the death rate at least for some strains at the beginning of the dynamics. This choice also ensures that all the resources are properly consumed and none of them grows indefinitely. In all simulations all the other parameters were set to the same values used in the main text.

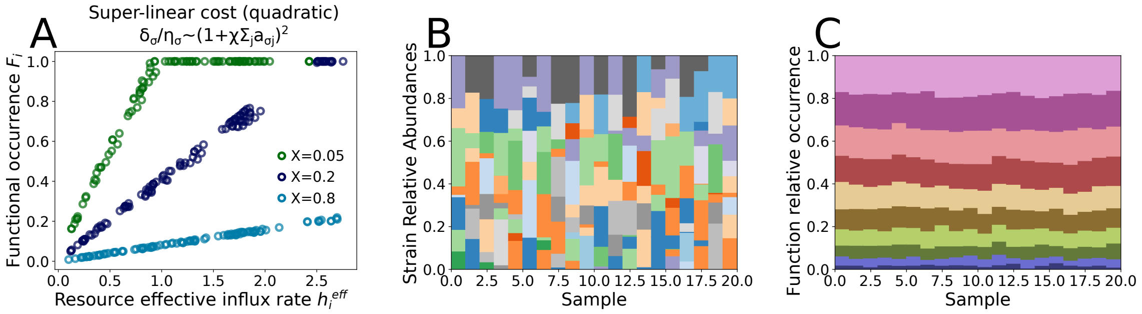

Fig J. Evolutionary outcomes under super-linear metabolic cost. All panels consider a quadratic cost ($g(z) = (1 + z)^2$). Panel A shows that the functional occurrences F_i^* depend on the effective resource influx rates h_i^{eff} in a similar fashion to what is observed for the linear metabolic cost (Fig 3 in the main text). Different points correspond both to different resources and different realizations of the intrinsic fitness values. Similar to the linear cost, increasing the value of the cost per resource χ decreases the number of core resources. The other panels show the taxonomic (panel B) and functional (panel C) composition of different communities evolved in independent environments, characterized by the same effective resource influx rate h_i^{eff} but different intrinsic fitness values ξ_σ. Panel B shows that the taxonomic composition varies widely across realizations, while the functional composition is much more stable and minimally affected by intrinsic-fitness variation (panel C). A color in panel B represents a strain, fully characterized by a given functional preference $a_{\sigma\cdot}$. Colors in panel C represent different functions. The overall qualitative picture confirms the results obtained in the main text for linear metabolic costs. In all simulations all the other parameters were set to the same values used in the main text. Panels B and C were obtained with $\chi = 0.5$.

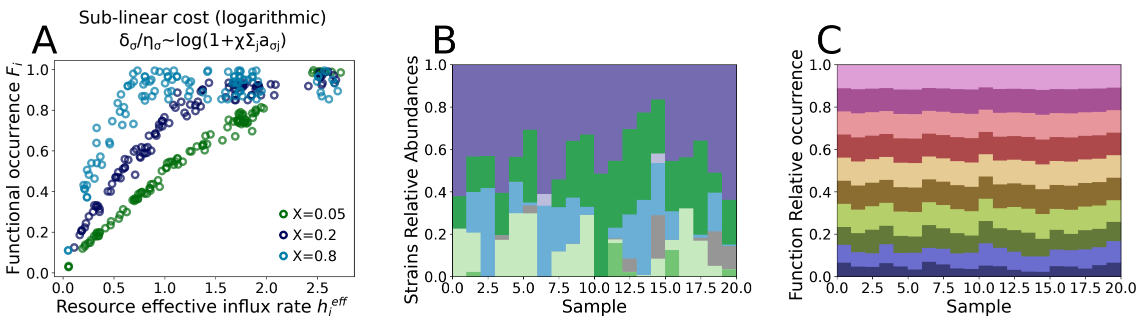

Fig K. Evolutionary outcomes under sub-linear metabolic cost. Same as Fig J but with a sub-linear metabolic cost. All panels consider the case of a logarithmic cost ($g(z) = \log(1 + z)$).

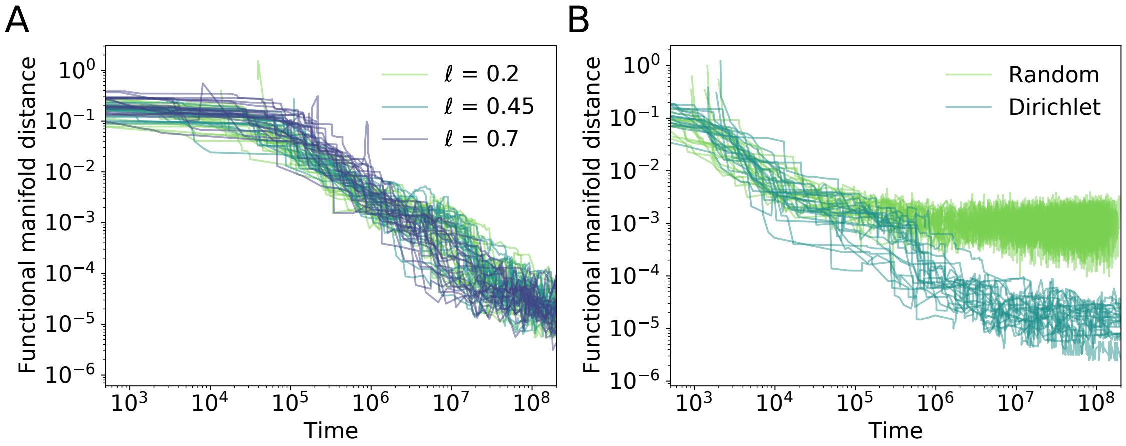

Fig L. Cross-feeding effect on the convergence to the functional manifold. The shape and intensity of cross-feeding affect neither the final distance from the manifold nor the path used to reach it. 20 realizations for every choice of the parameters are shown. A: effects of the amplitude of cross-feeding ℓ. Three values are considered: $\ell = 0.2$ in light green, $\ell = 0.45$ in green and $\ell = 0.7$ in blue. B: difference of convergence behavior in the presence of a random cross-feeding matrix and a Dirichlet-distributed matrix. The distance is defined in the main text. In all simulations all the other parameters were set to the same values used in the main text.
