# Supplementary material for: Eco-evolutionary dynamics lead to functionally robust and redundant communities
Source: PLoS Comput Biol. 2026 Jul 21;22(7):e1014437. doi: 10.1371/journal.pcbi.1014437 (PMC13432115; doi:10.1371/journal.pcbi.1014437)
Supplement: Attachment — Submitted filename: rebuttal.pdf [file pcbi.1014437.s003.pdf]

Response to Reviewers' Comments

Lorenzo Fant Iuri Macocco Jacopo Grilli

April 18, 2026

We thank the Editor and all three Reviewers for their careful reading of our manuscript and their constructive suggestions. We have thoroughly revised the manuscript to address all points raised. Below we provide detailed responses to each comment.

Editor's comments

Your manuscript has been evaluated by three experts of ecological modeling. All three reviewers are positive about your work and considered your research can make valuable contribution to the field. However, the reviewers have some concerns on model setups, parameter specification, and interpretation. They made constructive suggestions on models. I concur with their comments. The technical comments from the reviewers are extensive; nevertheless, I feel there is a good chance that you should be able to resolve those issues. Therefore, I am willing to consider your revision.

Your revision should address the specific points made by each reviewer. You should also send a cover letter indicating your responses to the comments and the changes you have made in the manuscript. If you disagree with a reviewer's point, please explain why. You should attempt to resolve the issues, rather than simply note that problems exist. If you choose to revise this submission, please provide a detailed response to each review, organized according to the points provided by the reviewers.

Reply: We thank the Editor for the positive assessment and the opportunity to revise. We have addressed all the specific points made by each reviewer, as detailed below. We have also addressed all journal requirements.

Reviewer 1

In the reviewed manuscript, the authors investigated properties of ecological system with multiple resources and multiple consumer strains (species) by constructing simulation model. In the model, they assumed a trade-off between individual survivorship and number of its available resource types. Based on the trade-off, they explored evolution of the resource utilization and the weight on the trade-off function (they refer it to "intrinsic fitness") in the per-capita growth rate in the consumers. According to the analysis, the authors showed functional robustness (i.e., stability of functional composition) and functional redundancy (i.e., variability of strain composition within the conserved function) in the simulated communities, which tend to correspond with observations in empirical studies. They also studied underlying mechanisms of the simulation results by investigating the simplified system analytically. I agree with the results of the model, although their novelties and implications seem limited.

Reply: We thank the reviewer for the careful reading and the constructive criticism. We address below each specific concern and hope to demonstrate that the novelty and implications of our work are substantial, particularly in providing a mechanistic explanation for functional redundancy that connects theoretical ecology with empirical observations in microbial communities.

Q 1.1 *(Major 1) Intrinsic fitness. (1) Definition: I feel that defining ξ_μ as the intrinsic fitness is very strange. Usually, the fitness corresponds with an offspring number of the individual. According to Eq. (1), ξ_μ affects a relative weight on the mortality that include the trade-off function, which seems inconsistent to the usual definition of fitness.*

Reply: We understand the Reviewer's concern regarding the terminology. In our model, ξ_μ can modulate the death rate (or equivalently, the maintenance cost) of strain μ. While "fitness" is often associated with offspring number, both reproduction and survival contribute to its value. To simplify, fitness is not simply the birth rate but the (expected) number of offspring in a lifetime (ratio of birth and death rate). Increasing death rate reduces the expected lifetime and therefore fitness.

Importantly, as apparent from equation 8, we can modulate "intrinsic fitness" equivalently by changing the death rate (by changing δ_σ) or the birth rate (modulating η_σ). These two alternatives (or any combination of the two) is equivalent in terms of model results. The reason is exactly that fitness effectively depends on the ratio of the two.

We clarified this distinction in the main text.

Q 1.2 *(Major 1) Intrinsic fitness. (2) The authors also assumed evolution of the intrinsic fitness,ξ_μ (p.4, l.103). I consider that when any restrictions are absent, ξ_μ must evolve toward 1, which can minimize the mortality. In the manuscript, however, I cannot find an explanation about constraints preventing ξ_μ from the increment.*

Reply: This is an important point. In our model, the intrinsic fitness of a new mutant is drawn *independently* from a fixed Gaussian distribution with mean zero and standard deviation ϵ, regardless of the parent's fitness value.Under this choice, fitness does not tend to increase to 1. We have now made this explicit in the main text (Section "Evolution of resource preferences and intrinsic fitness"). This choice is biologically motivated by the interpretation that intrinsic fitness differences arise from context-dependent factors — such as phage susceptibility, temperature adaptation, or other environmental variables — that do not accumulate progressively through selection.

The alternative scenario, where ξ is inherited from the parent with small perturbations (e.g., a staircase model as in Good et al. 2018, already cited in the manuscript), would indeed lead to a directional increase of ξ over time. We considered explicitely the staircase model and show in Fig. S12 that our results are robust even if fitness monotonically increase. We added a paragraph in the materials and methods to describe this scenario.

Q 1.3 *(Major 1) Intrinsic fitness. (3) Fluctuation: What is a relationship between trait value ξ_μ and its variation? Did the authors consider evolution of AVERAGE value of ξ_μ? If so, describe clearly that the evolvable trait is the AVERAGE of ξ_μ rather than ξ_μ itself*

Reply: We apologize for the confusion. There are two distinct scenarios in the paper that we have now more clearly separated in the revised text:

1. **Eco-evolutionary dynamics** (Figs. 2, 3): Each strain has a single, fixed ξ_μ value drawn at the time of the strain's appearance. There is no "average ξ_μ" per strain — ξ_μ is a fixed number for each strain, and it is the *net growth rate* that determines the strain's success.

2. **Longitudinal scenario** (Fig. 4C,D): The ξ_μ values of existing strains are made to fluctuate over time according to an Ornstein-Uhlenbeck process with autocorrelation timescale τ_{corr}. This is a separate model used to test the robustness of the functional attractor to temporal variation in fitness, not an evolutionary model. The fluctuation of ξ_μ mimics environmental variation (e.g., fluctuating temperature, changing phage communities) that affects strain fitness independently of their metabolic capabilities.

We have added a detailed description of the Ornstein-Uhlenbeck process for fitness fluctuations to the Materials and Methods section and clarified the distinction between the two scenarios in the main text.

Q 1.4 *(Major 2) Functional robustness: The authors evaluated the functional robustness by focusing on functional occurrence F_i that is the community consumption rate on resource type i, averaged across individuals. They showed that the functional occurrences are relatively constant even when the strain (species) composition varies within temporal dynamics or among simulation attempts (samples). However, the trend seems straightforward consequence of a balance between the total resource consumption and the resource supply. In the equilibrium (and the quasi-equilibrium following rapid resource dynamics), the total consumption rate on a specific resource type must correspond with its supply rate, which is not surprising. It is a simple consequence of the model structure, which did not provide implications. (The authors may recognize this point because they stated that F_i^* and N_i^* (Eqs. 2 and 3) are determined by the resource supply (p.6, l.162).*

Reply: We appreciate this comment, which allows us to clarify an important distinction. The Reviewer is correct that at ecological equilibrium, total resource consumption must balance supply. However, our result is substantially stronger than this observation, in two respects.

First, F_i is not the total consumption rate of resource i, but the *fraction of individuals* consuming it. The total consumption rate of resource i is $N \cdot F_i \cdot r_i(c_i^*)$. While supply-demand balance constrains the product $N \cdot F_i \cdot r_i(c_i^*)$, it does not, by itself, predict F_i independently. Our result shows that F_i is *uniquely* determined by environmental parameters (h_i^{eff}, χ), independently of which specific strains compose the community. This is a non-trivial prediction: with a different set of strains, one could have the same total consumption but a very different distribution of who consumes what.

Note that the supply-demand balance (constraining the product $F_i \cdot r_i(c_i^*)$) is equivalent to the functional composition F_i only if $r_i(c_i^*)$ is independent of i. This is exactly what happens when the functional attractor is reached: in that case resource concentration becomes independent of the supply. We believe that this is a non-trivial result.

Second, and more fundamentally, this independence from the species pool is a mathematical consequence of the metabolic trade-off, which causes the Lyapunov function governing the ecological dynamics to depend only on the total biomass N and the functional occurrences $\{F_i\}$, without reference to individual strain abundances (Eq. 8 in the revised manuscript). Without the trade-off, this property is lost.

To directly demonstrate the role of the species pool, we have added a new Supplementary Figure S9 showing that when the species pool is too limited (e.g., 5 strains for 15 resources, without evolution), the community cannot converge to the predicted functional composition, because the available diversity is insufficient to explore the functional space. In contrast, eco-evolutionary dynamics starting from the same small pool do converge, demonstrating the essential role of evolution in mimicking an infinite species pool.

We have substantially revised the main text to clarify this distinction and reference the new figure.

Q 1.5 *(Major 3) The authors emphasized that strain (species) composition significantly varies even under the functional robustness as illustrated in Figure 2, which was referred to the functional redundancy. The trend may include two aspects. In the early phase of evolutionary dynamics, there are many vacant niches. In this stage, the emergent strain could tend to have different trait from resident strains due to the colonization*

to the vacant niche, which is expected to cause a variation in the strain composition. On the other hand, near the equilibrium state (evolutionary steady state) after long elapsed time, the possible niches are already occupied. In this case, the emerging strains may necessarily have similar properties with the resident strains. With the similarity of properties, the strain replacement does not alter the community structure substantially, implying that the functional redundancy could be apparent. Thus, I consider that the functional redundancy should be evaluated by referring to properties of strains in the evolutionary system.

Reply: This is a thoughtful point. The Reviewer suggests that near equilibrium, replacing strains might be so similar to residents that the observed redundancy is "apparent" rather than genuine.

We can address this directly: the strains involved in turnover during the second phase (after functional convergence) typically belong to *different ecotypes* — they differ in their resource preference vectors $a_{\mu i}$, not just in their intrinsic fitness ξ_μ. Specifically, when one ecotype replaces another within the functional attractor, the two ecotypes often consume different subsets of resources. The aggregate functional composition F_i is preserved not because the replacing strain is functionally identical to the replaced one, but because the change in one strain's abundance is compensated by adjustments in other strains' abundances, maintaining the community-level F_i at its predicted value.

This is genuine redundancy: multiple distinct ecological strategies (ecotypes) can compose the community while maintaining the same functional profile. We have added a sentence in the revised text clarifying this point.

Q 1.6 *(Major 4) Numbers of persisting strains and resources. The results did not clearly indicate the number of persisting strains, although it seems to close to the number of resource types (i.e., 15 as the caption of Fig. 2) at each moment (typically after long elapsed time in simulations). This is consistent with a traditional prediction of species coexistence that the number of consumers cannot exceed the number of resource types in the resource–consumer system. Therefore, in the analyzed system, the number of resources would strongly restrict the coexistence of strains. Accordingly, I have a question. What type of resources were considered in the study? Is the number of resources (i.e., 15) realistic? The generality of the resource number should be also discussed.*

Reply: The Reviewer correctly observes that the number of coexisting strains is close to the number of resources R, consistent with the competitive exclusion principle. This is indeed expected and is a well-known property of consumer-resource models.

However, our key result is not about the number of coexisting strains, but about the functional composition: the *identity* and *relative abundance* of functions are uniquely predicted by environmental parameters, regardless of which specific strains fill those niches. The competitive exclusion principle sets an upper bound on the number of coexisting strains, but it does not predict the functional profile.

Regarding the biological interpretation: resources in our model are abstract and can be interpreted as distinct carbon sources, amino acids, vitamins, or any limiting nutrients. To demonstrate that our results are not specific to $R = 15$, we have added a new Supplementary Figure S11 showing that the functional attractor emerges for $R = 5, 10, 15$, and 25, with the same quantitative relationship between F_i and h_i^{eff}.

We have added a brief discussion of the biological interpretation of R and its realistic values in the revised manuscript.

Q 1.7 *Resource dynamics. (1) The main text did not include explanations about resource dynamic, which was described only in the Material and Methods section. I consider that the resource dynamics should be explained in the main text to some degree.*

Reply: We have added the resource dynamics equation (Eq. 3 in the revised manuscript) to the Results section, with a brief explanation of each term (external supply, consumption, and cross-feeding).

Q 1.8 *Resource dynamics. (2) In the Material and Methods section, the basal increasing rate of resource type i was denoted by $h_i(c_i)$ that is a function of the resource concentration, c_i (p.9, l.293). Subsequently, the authors proposed a specific assumption, $h_i(c_i) = h_i$, that is constant independently of the resource concentration (p.10, l.301). I consider that this is a critical assumption. If the h_i is a function of c_i, the system involves oscillating properties likewise the ordinary prey–predator model. The assumption should be stated*

more clearly in the main text. In addition, such oscillating dynamics may alter the model results. It seems better to discuss about it.

Reply: The Reviewer raises a valid point about the potential for oscillatory dynamics when resource supply depends on concentration. Our assumption $h_i(c_i) = h_i$ (constant external supply, independent of resource concentration) corresponds to a chemostat-like system and is standard in the consumer-resource literature (Tilman 1986, Posfai et al. 2017). This assumption is biologically appropriate for many microbial systems, including serial dilution and continuous culture experiments.

For the case of concentration-dependent resource supply (e.g., logistic growth $h_i(c_i) = r_i c_i$), the system could exhibit oscillatory dynamics, analogously to what happens in a Lotka-Volterra predatory-prey equation. These oscillations are neutral and structurally unstable. Note that the choice $h_i(c_i) = r_i c_i$ implies that, in absence of consumers, resources would grow exponentially without bounds. The addition of a carrying capacity (e.g., $h_i(c_i) = r_i c_i(1 - c_i/K_i)$) would remove the oscillations as it happens for Lotka-Volterra equations.

Moreover, under the timescale separation approximation — where resource dynamics are faster than population dynamics — the Lyapunov function structure is preserved and the equilibrium remains globally stable. We note that we already explore non-linear functional responses (Monod kinetics, Fig. S5) without affecting the results.

We have added a discussion of this assumption in the revised main text and Materials and Methods.

Q 1.9 *(Minor 1) Autocorrelation of fitness: I cannot find the explanation about the autocorrelation in the fitness fluctuation in the Methods.*

Reply: We thank the Reviewer for catching this omission. We have added a new subsection "Longitudinal fitness fluctuations" to the Materials and Methods, describing the Ornstein-Uhlenbeck process governing the temporal fluctuation of ξ_μ, including the autocorrelation timescale τ_{corr} and the diffusion coefficient.

Q 1.10 *(Minor 2) In Eq. (2), the "min" function should associate with parenthesis or curly bracket like Eq. (13).*

Reply: Corrected. Eq. (2) now uses curly brackets: $F_i^* = \min\{h_i^{eff}/(\chi N^*), 1\}$.

Reviewer 2

Functional redundancy is the important idea that many species can perform the same function, thereby allowing communities with different taxonomic compositions to have similar functional profiles. In this manuscript, the authors provide a mathematical model that can give rise to this phenomenon. The model is based on classical consumer-resource models with evolutionary dynamics. The authors elucidate the existence of functional attractors, which the system will converge to regardless of taxonomic composition. At these attractors, the functional occurrences are proportional to the effective resource influx, a form that can accomodate for the effects of cross-feeding. The attractors recapitulate the functional redundancy phenomenon both across samples and across time. However, the system does not converge to the functional attractors when intrinsic fitness effects are large enough such that species with high intrinsic fitness can survive regardless of their functional profiles. This manuscript asks a fundamental question and is timely, but I have questions about some of the results, as well as comments about the presentation.

Reply: We thank the Reviewer for the positive assessment and the insightful questions. We address each point below.

Q 2.1 *Many of the simulations spanned across* 10^9 *units of time, during which the functional attractor is reached as early as* $t \sim 10^7$*. What are the units here and how to understand these timescales? How do these timescales compare versus in real systems suspected to exhibit functional redundancy?*

Reply: Time in the model is measured in units of the inverse dilution rate (set to 1 without loss of generality). In chemostat-like microbial systems, dilution rates are typically $D \sim 0.1$–1 per hour, so one model time unit corresponds to approximately 1–10 hours.

With this mapping, convergence to the functional attractor at $t \sim 10^7$ corresponds to $\sim 10^3$–10^4 years. While this may seem long in absolute terms, we emphasize two points: (i) the convergence timescale is controlled by the mutation rate, which is a tunable parameter in the model — higher mutation rates lead to faster convergence (Supplementary Figure S4); (ii) the biologically relevant result is the *existence and universality* of the functional attractor, not the precise convergence time.

In real microbial populations with generation times of hours and large effective population sizes (10^8–10^{12}), the rate of adaptive mutations is very high, and the relevant metric is the number of successful invasions ($\sim$ hundreds) rather than absolute time. Furthermore, the functional redundancy observed in natural communities (Louca et al. 2016, 2018) is measured across spatially separated communities, not necessarily as a temporal convergence within a single community. Our model predicts that communities in similar environments converge to similar functional profiles, regardless of their evolutionary history.

We have added a new subsection "Timescale interpretation" in the Materials and Methods to discuss these issues.

Q 2.2 *Are there regimes where the functional attractor cannot be reached in a reasonable amount of time? How do the various modes of mutations affect the dynamics towards the functional attractor?*

Reply: Yes, there are several regimes where convergence to the functional attractor is impeded:

1. **Large intrinsic fitness differences** ($\epsilon/\chi \gg 1$): When fitness differences are large compared to the metabolic trade-off cost, the functional composition is dominated by the resource preferences of the fittest strains rather than by resource-driven selection. This is demonstrated in Supplementary Figures S1 ($\epsilon \sim 0.1$) and S2 ($\epsilon \sim 0.6$).

2. **Very low mutation rates**: With very low mutation rates, the phenotypic space is explored slowly and convergence takes proportionally longer (Supplementary Figure S4A). However, the attractor is still reached eventually.

3. **Weak metabolic trade-off** ($\chi \to 0$): Without a metabolic trade-off, there is no selection pressure favoring a specific functional composition, and the functional attractor does not exist. This follows directly from the Lyapunov function argument: when $\chi = 0$, the Lyapunov function no longer depends on the functional occurrences $\{F_i\}$.

The different mutation parameters (total rate U_{tot}, ratio U_-/U_+, relative contribution of horizontal gene transfer P_h/P_{dn}) affect the *speed* of convergence but not the *final state* (Supplementary Figure S4). We have added a new Results subsection "Robustness of the functional attractor" that discusses these findings systematically, integrating the supplementary figure results into the narrative.

Q 2.3 *The intrinsic fitness* $(1-\xi_\mu)$ *only affects the death rate term. What if it affects all components of the per capita growth rate in Eq. 1, including the resource consumption term? In this case, large noise should still destroy the functional attractor, but large compared to what?*

Reply: This is an excellent question that highlights a subtle but important modeling choice. There are three cases to consider:

1. ξ_μ **on the entire growth rate**: $g_\mu = (1+\xi_\mu)\cdot[\text{birth} - \text{death}]$. In this case, ξ_μ simply rescales the growth rate and cancels at equilibrium ($g_\mu = 0$), having *no effect* on community composition. This is trivial and uninteresting.

2. ξ_μ **on the death rate only** (our model): $g_\mu = \text{birth} - (1-\xi_\mu)\cdot\text{death}$. Fitness differences affect which strains win without modifying their metabolic capabilities. The functional attractor is preserved for small ϵ.

3. ξ_μ **on the birth rate (resource consumption)**: $g_\mu = (1+\xi_\mu)\cdot\text{birth} - \text{death}$. Here, ξ_μ modifies the birth rate only. This is equivalent in terms of model results to the above. The reason is that fitness effectively depends on the ratio of the two.

   We clarified this distinction in the main text.

Regarding "large compared to what": in our model, the relevant comparison is ϵ vs. χ. When $\epsilon/\chi \ll 1$, fitness differences are a small perturbation on the metabolic trade-off and the functional attractor is preserved. When $\epsilon/\chi \sim 1$ or larger, fitness effects dominate and the functional composition becomes dependent on the specific realization of fitness values.

We have added a paragraph in the Materials and Methods discussing this modeling choice.

Q 2.4 *More generally, I am somewhat confused about the novelty of the main findings and think that some reorganization can improve the manuscript. The fact that consumer-resource models can exhibit functional attractors is not new - Posfai et al (Ref 32) is a prominent example. Then, it seems straightforward that adding evolutionary dynamics that are slower than the ecological dynamics, which are still governed by a fixed environmental selection, will not affect the functional attractor. Thus, Fig 2 and Fig 4 are not very surprising in themselves. Fig 3 shows a nice relationship between functional occurence and effective resource influx - here again, my understanding is that a similar relationship has also been shown in Posfai et al but without cross-feeding. In this sense, much of the novel findings in this work are buried in the supplemental figures, which investigated how various model assumptions affect the functional attractor. However, being supplemental figures, these results are not discussed thoroughly enough in the text. Moreover, some important model details only appear in the Methods, which makes it even harder to extract the key findings. I suggest some rewriting to address these issues.*

Reply: We thank the Reviewer for this constructive suggestion. We have restructured the manuscript to better articulate the novelty and to integrate the supplementary results into the main narrative. Specifically:

Novelty relative to Posfai et al.: We now explicitly state in the Introduction and Discussion how our work extends beyond previous studies (Posfai et al., Tikhonov):

- We introduce *explicit eco-evolutionary dynamics* (mutation, horizontal gene transfer), showing that evolution effectively mimics an infinite species pool. Previous work assumed a fixed pool.
- We incorporate *cross-feeding*, which introduces effective resource influx rates h_i^{eff} that account for metabolic recycling. This extension is experimentally relevant (Goldford et al. 2018) and analytically non-trivial.

- We identify the *separation into two dynamical phases* (fast functional convergence + slow taxonomic turnover) and show that this provides a *mechanistic explanation for functional redundancy*, connecting the theoretical results with the empirical observations from metagenomics (Louca et al. 2016, 2018).

Integration of supplementary results: We have added a new Results subsection "Robustness of the functional attractor" that discusses all supplementary figures systematically.

Key model details: We have moved the resource dynamics equation into the main text and added the Ornstein-Uhlenbeck description and timescale interpretation to the Materials and Methods.

Reviewer 3

The paper is timely and insightful for microbial ecology and evolution. It addresses current challenges in identifying meaningful coarse-graining variables, and it highlights how resource availability and potential metabolic trade-offs shape community-level structure. Importantly, the authors convincingly show that small fitness differences alter taxon abundances without affecting functional abundances, reinforcing the distinction between taxonomic variability and functional stability. The modeling framework is well-described and the exploration of parameter space—including alternative cross-feeding structures, a range of mutation rates, and the extension to nonlinear trade-offs—is thorough and convincing. I found the manuscript enjoyable to read and conceptually rich. I recommend publication after minor revision. *This manuscript presents a theoretical framework for understanding the emergence of functional robustness and functional redundancy in microbial communities interacting through competition and cross-feeding. The authors show that eco-evolutionary trajectories converge rapidly to a functional attractor dictated solely by environmental conditions, even as taxonomic composition continues to drift in non-reproducible ways.*

The paper is timely and insightful for microbial ecology and evolution. It addresses current challenges in identifying meaningful coarse-graining variables, and it highlights how resource availability and potential metabolic trade-offs shape community-level structure. Importantly, the authors convincingly show that small fitness differences alter taxon abundances without affecting functional abundances, reinforcing the distinction between taxonomic variability and functional stability. The modeling framework is well-described and the exploration of parameter space—including alternative cross-feeding structures, a range of mutation rates, and the extension to nonlinear trade-offs—is thorough and convincing. I found the manuscript enjoyable to read and conceptually rich. I recommend publication after minor revision.

***Reply**: We thank the Reviewer for the encouraging assessment and the constructive suggestions. We address each point below.*

***Q 3.1** (Major) Clarification and deeper explanation of Fig. 4 (Main suggestion): The explanation accompanying Fig. 4 is somewhat shallow.Differences in initial fitness will of course give different community composition. Mutations will modify the intrinsic fitness, and I don't understand what is the fundamental difference between fig 2 A and 4C, other than the linear vs log timescale. In this sense: why choosing the timescale of 4c? From 2C it seems that the dymamics becomes ever slower, so it is not clear to me which is the right turnover timescale to look at in linear scale. Moreover, the explanation in line 196 discusses autocorrelation timescales, but cites no figure in which there is a quantitative analysis of autocorrelation time.*

***Reply**: We agree that the original explanation of Fig. 4 was insufficiently detailed. The fundamental difference between Fig. 2A and Fig. 4C is:*

- ***Fig. 2A** shows the* assembly *of a community from a clonal population. Both the functional and taxonomic composition are changing during the initial transient (the first phase of the eco-evolutionary dynamics). The community is "building up" its functional profile through niche-filling invasions. A logarithmic time axis is used because the dynamics span multiple orders of magnitude, from the rapid initial assembly to the slow subsequent evolution.*
- ***Fig. 4C** shows a community that has* already reached *the functional attractor. The taxonomic turnover depicted in Fig. 4C is driven entirely by the fluctuating fitness landscape (Ornstein-Uhlenbeck process on ξ_μ), not by niche filling. The functional composition stays at the attractor throughout. A linear time axis is used because the turnover dynamics are* stationary *— there is no transient requiring a logarithmic scale. The linear timescale highlights the ongoing, roughly constant-rate taxonomic replacement.*

Regarding the autocorrelation analysis: we have added a new Supplementary Figure S10 that quantifies the autocorrelation of both taxonomic and functional composition as a function of time lag, for different values of the fitness autocorrelation timescale τ_{corr}. This figure confirms that functional similarity remains close to 1 regardless of τ_{corr}, while taxonomic similarity decays at a rate controlled by τ_{corr}.

We have substantially revised the text accompanying Fig. 4 to include these explanations and reference the new figure.

Q 3.2 *(Minor) Explanation of* h_i^{eff} *in the main text.*

Reply*: We have added an explicit formula for* h_i^{eff} *where it is first introduced in the Results section:* $h_i^{eff} = (1-\ell)\sum_j B_{ij}h_j w_j$ *where* $B = (I - \ell D)^{-1}$.

Q 3.3 *(Minor) Line 88: Missing comma after "linear".*

Reply*: Corrected: "linear, sub-linear, and super-linear".*

Q 3.4 *(Minor) Some panels of Fig 2 should be cited earlier.*

Reply*: We now cite Fig. 2A when describing strain abundance dynamics and Fig. 2B,C when discussing functional occurrences and biomass convergence.*

Q 3.5 *(Minor) Line 158: The two sentences here express the same idea.*

Reply*: We have removed the redundant sentence.*

Q 3.6 *(Minor) Line 173: The necessity of this sentence is unclear [...] I recommend removing "Nevertheless".*

Reply*: We have revised this passage to avoid redundancy and removed the problematic "Nevertheless" construction.*

Q 3.7 *(Minor) Figure 1: The color gradient for the metabolic cost in the legend suggests meaning that it does not actually have. Consider replacing "This demonstrates how..." with "This can be interpreted as..."*

Reply*: We have replaced "This demonstrates how" with "This can be interpreted as" in the Figure 1 caption. We will review the color gradient in the legend for the final production version.*

Q 3.8 *(Minor) Figure 2: I could not figure out how to read the 'three different realizations of the system'.*

Reply*: We have expanded the Figure 2 caption to clarify: "Each colored line represents a different strain; strains appear and disappear as invasions and extinctions occur" (Panel A) and "three different realizations [...] distinguished by line style. Different colors represent different resources. The three realizations converge to the same functional profile despite starting from different initial conditions" (Panel B).*

Q 3.9 *(Minor) Figure 3, panel (b): the legend should read "average resource income" rather than "resource input."*

Reply*: Corrected in the caption.*

Q 3.10 *(Minor) Figure 4 caption: Replace "allow to demonstrate" with "demonstrates"; choose consistent terminology; describe 'static noise' and 'dynamically varying noise'.*

Reply*: We have rewritten the Figure 4 caption. "Allow to demonstrate" has been replaced with "demonstrate". We use "strains" consistently throughout. We now explicitly define "static" (constant intrinsic fitness values, varying across communities) and "dynamically varying" (intrinsic fitness fluctuating over time via an Ornstein-Uhlenbeck process) scenarios.*
