# Supplementary material for: Eco-evolutionary dynamics lead to functionally robust and redundant communities
Source: PLoS Comput Biol. 2026 Jul 21;22(7):e1014437. doi: 10.1371/journal.pcbi.1014437 (PMC13432115; doi:10.1371/journal.pcbi.1014437)
Supplement: Peer review history

# Peer review: Decision Letter 0

**DOI:** 10.1371/journal.pcbi.1014437.r001

**Authors:** Chih-hao Hsieh

16 Feb 2026

-->PCOMPBIOL-D-26-00048

Eco-evolutionary dynamics lead to functionally robust and redundant communities

PLOS Computational Biology

Dear Dr. Grilli,

Thank you for submitting your manuscript to PLOS Computational Biology. After careful consideration, we feel that it has merit but does not fully meet PLOS Computational Biology’s publication criteria as it currently stands. Therefore, we invite you to submit a revised version of the manuscript that addresses the points raised during the review process.

Please submit your revised manuscript by Apr 18 2026 11:59PM. If you will need more time than this to complete your revisions, please reply to this message or contact the journal office at ploscompbiol@plos.org. When you're ready to submit your revision, log on to https://www.editorialmanager.com/pcompbiol/ and select the 'Submissions Needing Revision' folder to locate your manuscript file.

Please include the following items when submitting your revised manuscript:

* A letter that responds to each point raised by the editor and reviewer(s). You should upload this letter as a separate file labeled 'Response to Reviewers'. This file does not need to include responses to formatting updates and technical items listed in the 'Journal Requirements' section below.

* A marked-up copy of your manuscript that highlights changes made to the original version. You should upload this as a separate file labeled 'Revised Manuscript with Track Changes'.

* An unmarked version of your revised paper without tracked changes. You should upload this as a separate file labeled 'Manuscript'.

If you would like to make changes to your financial disclosure, competing interests statement, or data availability statement, please make these updates within the submission form at the time of resubmission. Guidelines for resubmitting your figure files are available below the reviewer comments at the end of this letter

We look forward to receiving your revised manuscript.

Kind regards,

Chih-hao Hsieh, Ph.D.

Guest Editor

PLOS Computational Biology

Feng Fu

Section Editor

PLOS Computational Biology

**Additional Editor Comments:**

Your manuscript has been evaluated by three experts of ecological modeling. All three reviewers are positive about your work and considered your research can make valuable contribution to the field. However, the reviewers have some concerns on model setups, parameter specification, and interpretation. They made constructive suggestions on models. I concur with their comments. The technical comments from the reviewers are extensive; nevertheless, I feel there is a good chance that you should be able to resolve those issues. Therefore, I am willing to consider your revision.

Your revision should address the specific points made by each reviewer. You should also send a cover letter indicating your responses to the comments and the changes you have made in the manuscript. If you disagree with a reviewer's point, please explain why. You should attempt to resolve the issues, rather than simply note that problems exist. If you choose to revise this submission, please provide a detailed response to each review, organized according to the points provided by the reviewers.

Specific comments from reviewers are appended below or in attached files.

**Journal Requirements:**

If the reviewer comments include a recommendation to cite specific previously published works, please review and evaluate these publications to determine whether they are relevant and should be cited. There is no requirement to cite these works unless the editor has indicated otherwise.

1) Please ensure that the CRediT author contributions listed for every co-author are completed accurately and in full.

At this stage, the following Authors/Authors require contributions: Lorenzo Fant, Iuri Macocco, and Jacopo Grilli. Please ensure that the full contributions of each author are acknowledged in the "Add/Edit/Remove Authors" section of our submission form.

The list of CRediT author contributions may be found here: https://journals.plos.org/ploscompbiol/s/authorship#loc-author-contributions

2) We ask that a manuscript source file is provided at Revision. Please upload your manuscript file as a .doc, .docx, .rtf or .tex. If you are providing a .tex file, please upload it under the item type u2018LaTeX Source Fileu2019 and leave your .pdf version as the item type u2018Manuscriptu2019.

3) Please provide an Author Summary. This should appear in your manuscript between the Abstract (if applicable) and the Introduction, and should be 150-200 words long. The aim should be to make your findings accessible to a wide audience that includes both scientists and non-scientists. Sample summaries can be found on our website under Submission Guidelines:

https://journals.plos.org/ploscompbiol/s/submission-guidelines#loc-parts-of-a-submission

4) Please upload all main figures as separate Figure files in .tif or .eps format. For more information about how to convert and format your figure files please see our guidelines:

https://journals.plos.org/ploscompbiol/s/figures

5) We notice that your supplementary Figures are included in the manuscript file. Please remove them and upload them with the file type 'Supporting Information'. Please ensure that each Supporting Information file has a legend listed in the manuscript after the references list.

6) Some material included in your submission may be copyrighted. According to PLOSu2019s copyright policy, authors who use figures or other material (e.g., graphics, clipart, maps) from another author or copyright holder must demonstrate or obtain permission to publish this material under the Creative Commons Attribution 4.0 International (CC BY 4.0) License used by PLOS journals. Please closely review the details of PLOSu2019s copyright requirements here: PLOS Licenses and Copyright. If you need to request permissions from a copyright holder, you may use PLOS's Copyright Content Permission form.

Please respond directly to this email and provide any known details concerning your material's license terms and permissions required for reuse, even if you have not yet obtained copyright permissions or are unsure of your material's copyright compatibility. Once you have responded and addressed all other outstanding technical requirements, you may resubmit your manuscript within Editorial Manager.

Potential Copyright Issues:

i) Figure 1. Please confirm whether you drew the images / clip-art within the figure panels by hand. If you did not draw the images, please provide (a) a link to the source of the images or icons and their license / terms of use; or (b) written permission from the copyright holder to publish the images or icons under our CC BY 4.0 license. Alternatively, you may replace the images with open source alternatives. See these open source resources you may use to replace images / clip-art:

- https://commons.wikimedia.org

- https://openclipart.org/.

7) Please amend your detailed Financial Disclosure statement. This is published with the article. It must therefore be completed in full sentences and contain the exact wording you wish to be published.

1) State the initials, alongside each funding source, of each author to receive each grant. For example: "This work was supported by the National Institutes of Health (####### to AM; ###### to CJ) and the National Science Foundation (###### to AM)."

2) State what role the funders took in the study. If the funders had no role in your study, please state: "The funders had no role in study design, data collection and analysis, decision to publish, or preparation of the manuscript."

3) If any authors received a salary from any of your funders, please state which authors and which funders..

If you did not receive any funding for this study, please simply state: u201cThe authors received no specific funding for this work.u201d

8) Please send a completed 'Competing Interests' statement, including any COIs declared by your co-authors. If you have no competing interests to declare, please state "The authors have declared that no competing interests exist". Otherwise please declare all competing interests beginning with the statement "I have read the journal's policy and the authors of this manuscript have the following competing interests"

**Reviewers' comments:**

Reviewer's Responses to Questions

**Comments to the Authors:**

**Please note here if the review is uploaded as an attachment.**

Reviewer #1: Review of "Eco-evolutionary dynamics lead to functionally robust and redundant communities" (MS#: PCOMPBIOL-D-26-00048) by Lorenzo Fant, Iuri Macocco, and Jacopo Grilli,

In the reviewed manuscript, the authors investigated properties of ecological system with multiple resources and multiple consumer strains (species) by constructing simulation model. In the model, they assumed a trade-off between individual survivorship and number of its available resource types. Based on the trade-off, they explored evolution of the resource utilization and the weight on the trade-off function (they refer it to "intrinsic fitness") in the per-capita growth rate in the consumers. According to the analysis, the authors showed functional robustness (i.e., stability of functional composition) and functional redundancy (i.e., variability of strain composition within the conserved function) in the simulated communities, which tend to correspond with observations in empirical studies. They also studied underlying mechanisms of the simulation results by investigating the simplified system analytically. I agree with the results of the model, although their novelties and implications seem limited.

Major issues

1. Intrinsic fitness

(1) Definition

I feel that defining ξ_μ as the intrinsic fitness (p.4, l.97) is very strange. Usually, the fitness corresponds with an offspring number of the individual. According to Eq. (1), ξ_μ affects a relative weight on the mortality that include the trade-off function, which seems inconsistent to the usual definition of fitness.

(2) Evolution

The authors also assumed evolution of the intrinsic fitness,ξ_μ (p.4, l.103). I consider that when any restrictions are absent, ξ_μ must evolve toward 1, which can minimize the mortality. In the manuscript, however, I cannot find an explanation about constraints preventing ξ_μ from the increment.

(3) Fluctuation

Furthermore, the authors considered the fluctuation of ξ_μ (p.6, 188). What is a relationship between trait value ξ_μ and its variation? Did the authors consider evolution of AVERAGE value of ξ_μ? If so, describe clearly that the evolvable trait is the AVERAGE of ξ_μ rather than ξ_μ itself.

2. Functional robustness

The authors evaluated the functional robustness by focusing on functional occurrence F_i that is the community consumption rate on resource type i, averaged across individuals. They showed that the functional occurrences are relatively constant even when the strain (species) composition varies within temporal dynamics or among simulation attempts (samples). However, the trend seems straightforward consequence of a balance between the total resource consumption and the resource supply. In the equilibrium (and the quasi-equilibrium following rapid resource dynamics), the total consumption rate on a specific resource type must correspond with its supply rate, which is not surprising. It is a simple consequence of the model structure, which did not provide implications.

(The authors may recognize this point because they stated that F*_i and N*_i (Eqs. 2 and 3) are determined by the resource supply (p.6, l.162).)

2. Functional redundancy

The authors emphasized that strain (species) composition significantly varies even under the functional robustness as illustrated in Figure 2, which was referred to the functional redundancy. The trend may include two aspects.

In the early phase of evolutionary dynamics, there are many vacant niches. In this stage, the emergent strain could tend to have different trait from resident strains due to the colonization to the vacant niche, which is expected to cause a variation in the strain composition.

On the other hand, near the equilibrium state (evolutionary steady state) after long elapsed time, the possible niches are already occupied. In this case, the emerging strains may necessarily have similar properties with the resident strains. With the similarity of properties, the strain replacement does not alter the community structure substantially, implying that the functional redundancy could be apparent. Thus, I consider that the functional redundancy should be evaluated by referring to properties of strains in the evolutionary system.

3. Numbers of persisting strains and resources

The results did not clearly indicate the number of persisting strains, although it seems to close to the number of resource types (i.e., 15 as the caption of Fig. 2) at each moment (typically after long elapsed time in simulations). This is consistent with a traditional prediction of species coexistence that the number of consumers cannot exceed the number of resource types in the resource–consumer system. Therefore, in the analyzed system, the number of resources would strongly restrict the coexistence of strains. Accordingly, I have a question. What type of resources were considered in the study? Is the number of resources (i.e., 15) realistic? The generality of the resource number should be also discussed.

4. Resource dynamics

(1) The main text

The main text did not include explanations about resource dynamic, which was described only in the Material and Methods section. I consider that the resource dynamics should be explained in the main text to some degree.

(2) Basal increasing rate of resource

In the Material and Methods section, the basal increasing rate of resource type i was denoted by h_i(c_i) that is a function of the resource concentration, c_i (p.9, l.293). Subsequently, the authors proposed a specific assumption, h_i(c_i) = h_i, that is constant independently of the resource concentration (p.10, l.301). I consider that this is a critical assumption. If the h_i is a function of c_i, the system involves oscillating properties likewise the ordinary prey–predator model. The assumption should be stated more clearly in the main text. In addition, such oscillating dynamics may alter the model results. It seems better to discuss about it.

Minor issues

(1) The authors mentioned (temporal?) fluctuation of fitness with autocorrelation, which was referred to "Material and Method" (p.7, l.196). However, I cannot find the explanation about the autocorrelation in the fitness fluctuation in the section.

(2) In Eq. (2), the "min" function should associate with parenthesis or curly bracket like Eq. (13).

Reviewer #2: Functional redundancy is the important idea that many species can perform the same function, thereby allowing communities with different taxonomic compositions to have similar functional profiles. In this manuscript, the authors provide a mathematical model that can give rise to this phenomenon. The model is based on classical consumer-resource models with evolutionary dynamics. The authors elucidate the existence of functional attractors, which the system will converge to regardless of taxonomic composition. At these attractors, the functional occurrences are proportional to the effective resource influx, a form that can accomodate for the effects of cross-feeding. The attractors recapitulate the functional redundancy phenomenon both across samples and across time. However, the system does not converge to the functional attractors when intrinsic fitness effects are large enough such that species with high intrinsic fitness can survive regardless of their functional profiles. This manuscript asks a fundamental question and is timely, but I have questions about some of the results, as well as comments about the presentation.

- Many of the simulations spanned across 10^9 units of time, during which the functional attractor is reached as early as t ~ 10^7 (Fig 2). In other words, the system is in the functional attractor for the vast majority of the simulations. What are the units here and how to understand these timescales? How do these timescales compare versus in real systems suspected to exhibit functional redundancy?

- Related to the above question, the mutation rates, the probability of horizontal gene transfer, and other evolutionary dynamics parameters all seem to affect the rate of convergence towards the functional attractor (Supp Fig S4). Are there regimes where the functional attractor cannot be reached in a reasonable amount of time? How do the various modes of mutations affect the dynamics towards the funtional attractor?

- The intrinsic fitness (1 - xi_mu) only affects the death rate term. What if it affects all components of the per capita growth rate in Eq. 1, including the resource consumption term? In this case, large noise should still destroy the functional attractor, but large compared to what?

- More generally, I am somewhat confused about the novelty of the main findings and think that some reorganization can improve the manuscript. The fact that consumer-resource models can exhibit functional attractors is not new - Posfai et al (Ref 32) is a prominent example. Then, it seems straightforward that adding evolutionary dynamics that are slower than the ecological dynamics, which are still governed by a fixed environmental selection, will not affect the functional attractor. Thus, Fig 2 and Fig 4 are not very surprising in themselves. Fig 3 shows a nice relationship between functional occurence and effective resource influx - here again, my understanding is that a similar relationship has also been shown in Posfai et al but without cross-feeding. In this sense, much of the novel findings in this work are buried in the supplemental figures, which investigated how various model assumptions affect the functional attractor. However, being supplemental figures, these results are not discussed thoroughly enough in the text. Moreover, some important model details only appear in the Methods, which makes it even harder to extract the key findings. I suggest some rewriting to address these issues.

Reviewer #3: This manuscript presents a theoretical framework for understanding the emergence of functional robustness and functional redundancy in microbial communities interacting through competition and cross‑feeding. The authors show that eco‑evolutionary trajectories converge rapidly to a functional attractor dictated solely by environmental conditions, even as taxonomic composition continues to drift in non‑reproducible ways.

The paper is timely and insightful for microbial ecology and evolution. It addresses current challenges in identifying meaningful coarse‑graining variables, and it highlights how resource availability and potential metabolic trade‑offs shape community‑level structure. Importantly, the authors convincingly show that small fitness differences alter taxon abundances without affecting functional abundances, reinforcing the distinction between taxonomic variability and functional stability.

The modeling framework is well‑described and the exploration of parameter space—including alternative cross‑feeding structures, a range of mutation rates, and the extension to nonlinear trade‑offs—is thorough and convincing. I found the manuscript enjoyable to read and conceptually rich.

I recommend publication after minor revision.

Major Comment

- Clarification and deeper explanation of Fig. 4 (Main suggestion):

The explanation accompanying Fig. 4 is somewhat shallow.Differences in initial fitness will of course give different community composition. Mutations will modify the intrinsic fitness, and I don’t understand what is the fundamental difference between fig 2 A and 4C, other than the linear vs log timescale. In this sense: why choosing the timescale of 4c? From 2C it seems that the dymamics becomes ever slower, so it is not clear to me which is the right turnover timescale to look at in linear scale. Moreover, the explanation in line 196 discusses autocorrelation timescales, but cites no figure in which there is a quantitative analysis of autocorrelation time.

Minor Comments

- Explanation of h_i^eff in the main text: it could be helpful to briefly explain in the Results section how the effective availability terms are computed. The manuscript is readable without this, so I leave it to the authors’ discretion.

- Line 88: Missing comma after “linear”.

- Some panels of Fig 2 should be cited earlier, perhaps in paragraphs starting in lines 128 and 130, where the properties of typical times series are described. Also, the claim in line 133 on reproducibility across different realizations should cite a figure panel that supports the claim

- Line 158: The two sentences here express the same idea; I suggest removing one.

- Line 173: The necessity of this sentence is unclear, as the previous one already summarizes the main point effectively. If kept, I recommend removing “Nevertheless,” which does not seem appropriate.

- Figure 1: The color gradient for the metabolic cost in the legend suggests meaning that it does not actually have. Consider adjusting the cartoon or legend to avoid confusion. In the caption, consider replacing “This demonstrates how…” with “This can be interpreted as…” for accuracy.

- Figure 2: I could not figure out how to read the 'three different realizations of the system' mentioned in the caption. Describe better what each line is representing.

- Figure 3: In panel (b), the legend should read “average resource income” rather than “resource input.”

- Figure 4, caption: Replace “allow to demonstrate” with “demonstrates” which I think is what the authors mean. Choose either “taxonomies” or “populations” and use it consistently. For a more comprehensive caption, the authors could describe 'static noise' and 'dynamically varying noise'. The two categories are understandable from the main text, but I think that they are not really defined by their name (static or dynamic).

**********

**Have the authors made all data and (if applicable) computational code underlying the findings in their manuscript fully available?**

The PLOS Data policy requires authors to make all data and code underlying the findings described in their manuscript fully available without restriction, with rare exception (please refer to the Data Availability Statement in the manuscript PDF file). The data and code should be provided as part of the manuscript or its supporting information, or deposited to a public repository. For example, in addition to summary statistics, the data points behind means, medians and variance measures should be available. If there are restrictions on publicly sharing data or code —e.g. participant privacy or use of data from a third party—those must be specified.

Reviewer #1: Yes

Reviewer #2: None

Reviewer #3: Yes

**********

PLOS authors have the option to publish the peer review history of their article (what does this mean?). If published, this will include your full peer review and any attached files.

If you choose “no”, your identity will remain anonymous but your review may still be made public.

**Do you want your identity to be public for this peer review?**  For information about this choice, including consent withdrawal, please see our Privacy Policy.

Reviewer #1: No

Reviewer #2: No

Reviewer #3: No

**Figure resubmission:**

While revising your submission, we strongly recommend that you use PLOS’s NAAS tool (https://ngplosjournals.pagemajik.ai/artanalysis) to test your figure files. NAAS can convert your figure files to the TIFF file type and meet basic requirements (such as print size, resolution), or provide you with a report on issues that do not meet our requirements and that NAAS cannot fix.-->

After uploading your figures to PLOS’s NAAS tool - https://ngplosjournals.pagemajik.ai/artanalysis, NAAS will process the files provided and display the results in the "Uploaded Files" section of the page as the processing is complete. If the uploaded figures meet our requirements (or NAAS is able to fix the files to meet our requirements), the figure will be marked as "fixed" above. If NAAS is unable to fix the files, a red "failed" label will appear above. When NAAS has confirmed that the figure files meet our requirements, please download the file via the download option, and include these NAAS processed figure files when submitting your revised manuscript.

**Reproducibility:**

To enhance the reproducibility of your results, we recommend that authors of applicable studies deposit laboratory protocols in protocols.io, where a protocol can be assigned its own identifier (DOI) such that it can be cited independently in the future. Additionally, PLOS ONE offers an option to publish peer-reviewed clinical study protocols. Read more information on sharing protocols at https://plos.org/protocols?utm_medium=editorial-email&utm_source=authorletters&utm_campaign=protocols

# Author comment: Author response to Decision Letter 1

**DOI:** 10.1371/journal.pcbi.1014437.r002

7 May 2026

Attachment Submitted filename: rebuttal.pdf

# Peer review: Decision Letter 1

**DOI:** 10.1371/journal.pcbi.1014437.r003

**Authors:** Chih-hao Hsieh

14 Jun 2026

Dear Dr Grilli,

We are pleased to inform you that your manuscript 'Eco-evolutionary dynamics lead to functionally robust and redundant communities' has been provisionally accepted for publication in PLOS Computational Biology.

Before your manuscript can be formally accepted you will need to complete some formatting changes, which you will receive in a follow up email. A member of our team will be in touch with a set of requests.

Please note that your manuscript will not be scheduled for publication until you have made the required changes, so a swift response is appreciated.

IMPORTANT: The editorial review process is now complete. PLOS will only permit corrections to spelling, formatting or significant scientific errors from this point onwards. Requests for major changes, or any which affect the scientific understanding of your work, will cause delays to the publication date of your manuscript.

Should you, your institution's press office or the journal office choose to press release your paper, you will automatically be opted out of early publication. We ask that you notify us now if you or your institution is planning to press release the article. All press must be co-ordinated with PLOS.

Thank you again for supporting Open Access publishing; we are looking forward to publishing your work in PLOS Computational Biology.

Best regards,

Chih-hao Hsieh, Ph.D.

Guest Editor

PLOS Computational Biology

Feng Fu

Section Editor

PLOS Computational Biology

***********************************************************

Reviewer's Responses to Questions

**Comments to the Authors:**

**Please note here if the review is uploaded as an attachment.**

Reviewer #2: The authors have thoroughly addressed my previous comments. I am happy to recommend publication.

Reviewer #3: In my opinion, the authors have appropriately addressed the comments by the referees in the revised version of the text.

**********

**Have the authors made all data and (if applicable) computational code underlying the findings in their manuscript fully available?**

The PLOS Data policy requires authors to make all data and code underlying the findings described in their manuscript fully available without restriction, with rare exception (please refer to the Data Availability Statement in the manuscript PDF file). The data and code should be provided as part of the manuscript or its supporting information, or deposited to a public repository. For example, in addition to summary statistics, the data points behind means, medians and variance measures should be available. If there are restrictions on publicly sharing data or code —e.g. participant privacy or use of data from a third party—those must be specified.

Reviewer #2: None

Reviewer #3: Yes

**********

PLOS authors have the option to publish the peer review history of their article (what does this mean?). If published, this will include your full peer review and any attached files.

If you choose “no”, your identity will remain anonymous but your review may still be made public.

**Do you want your identity to be public for this peer review?**  For information about this choice, including consent withdrawal, please see our Privacy Policy.

Reviewer #2: No

Reviewer #3: No

# Editor report: Acceptance letter

**DOI:** 10.1371/journal.pcbi.1014437.r004

**Authors:** Chih-hao Hsieh

PCOMPBIOL-D-26-00048R1

Eco-evolutionary dynamics lead to functionally robust and redundant communities

Dear Dr Grilli,

I am pleased to inform you that your manuscript has been formally accepted for publication in PLOS Computational Biology. Your manuscript is now with our production department and you will be notified of the publication date in due course.

The corresponding author will soon be receiving a typeset proof for review, to ensure errors have not been introduced during production. Please review the PDF proof of your manuscript carefully, as this is the last chance to correct any errors. Please note that major changes, or those which affect the scientific understanding of the work, will likely cause delays to the publication date of your manuscript.

Soon after your final files are uploaded, unless you have opted out, the early version of your manuscript will be published online. The date of the early version will be your article's publication date. The final article will be published to the same URL, and all versions of the paper will be accessible to readers.

For Research, Software, and Methods articles, you will receive an invoice from PLOS for your publication fee after your manuscript has reached the completed accept phase. If you receive an email requesting payment before acceptance or for any other service, this may be a phishing scheme. Learn how to identify phishing emails and protect your accounts at https://explore.plos.org/phishing.

Thank you again for supporting PLOS Computational Biology and open-access publishing. We are looking forward to publishing your work!

With kind regards,

Janani Seenivasan

PLOS Computational Biology | Carlyle House, Carlyle Road, Cambridge CB4 3DN | United Kingdom ploscompbiol@plos.org | Phone +44 (0) 1223-442824 | ploscompbiol.org | @PLOSCompBiol
